# Genomic reconstruction of upland cotton domestication uncovers staged selection, gene flow, and flowering-time adaptation

**DOI:** 10.1073/pnas.2601246123

**Published:** 2026-06-22

**Authors:** Yanchao Xu, Xiaoyan Cai, Zhongli Zhou, Damar Lopez-Arredondo, Yuqing Hou, Jie Zheng, Hongge Li, Gaofei Sun, Dingsha Jin, Panhong Dai, Yangyang Wei, Yuling Liu, Pengtao Li, Qiankun Liu, Heng Wang, Runrun Sun, Lijie Li, Xiaoping Pan, Kunbo Wang, Xiongming Du, Guoli Song, Baohong Zhang, Luis Rafael Herrera-Estrella, Shoupu He, Fang Liu, Renhai Peng

**Affiliations:** ^a^ State Key Laboratory of Cotton Bio-breeding and Integrated Utilization, Institute of Cotton Research, Chinese Academy of Agricultural Sciences, Anyang 455000, China; ^b^ Anyang Institute of Technology, Anyang 455000, China; ^c^ National Nanfan Research Institute (Sanya), Chinese Academy of Agricultural Sciences, Sanya 572024, China; ^d^ Department of Plant and Soil Science, Institute of Genomics for Crop Abiotic Stress Tolerance, Texas Tech University, Lubbock, TX 79409; ^e^Sanya Research Institute, Hainan Academy of Agricultural Sciences, Sanya 572024, China; ^f^ Department of Biology, East Carolina University, Greenville, NC 27858; ^g^ Henan Institute of Science and Technology, Xinxiang, Henan 453000, China; ^h^ Unidad de Genómica Avanzada del Centro de Investigación y de Estudios Avanzados del Instituto Politécnico Nacional, Irapuato, Guanajuato 36824, Mexico; ^i^ School of Life Science, School of Agriculture and Biomanufacturing, Zhengzhou University, Zhengzhou 450001, China

**Keywords:** crop domestication, cotton pangenome, domestication traits, domestication trajectory, cotton fiber

## Abstract

Upland cotton (*Gossypium hirsutum* L.) makes up about 90% of global cotton farming. Despite its importance, the origins and domestication history of upland cotton remain poorly understood. Here, we present a high-density pan-genome variation map constructed from 2,910 cotton accessions, including 440 newly sequenced *G. hirsutum* landraces. Our pan-genome analysis indicates that modern upland cotton most probably originated from a single domestication and underwent three major stages. We also identified several genomic signatures associated with agriculturally important traits, including photoperiod sensitivity, fiber properties, and seed yield, which map to candidate loci *GhTOFD06*, *GhFLD11,* and *GhSID05,* respectively, through a genome-wide association study (GWAS) and VIGS knockdown. During the third domestication stage (D3), selective pressures favored genes, such as GhTOFD06, a homolog of Arabidopsis COP9 signalosome complex subunit 5b (CSN5B), that control photoperiod-regulated flowering, thereby accelerating cotton domestication and facilitating upland cotton’s latitudinal expansion. Notably, functional validation via gene silencing showed that knockdown of *GhSID05* reduced single seed weight by 11.41%, confirming its pivotal role in seed yield regulation. Additionally, we revealed natural gene flow between *G. hirsutum* and *G. barbadense*, which has significantly enriched the genetic diversity of the *G. hirsutum* gene pool and may have contributed favorable alleles for improving modern upland cotton. Our study provides a comprehensive understanding of the genomic evolution of *G. hirsutum* and valuable genetic resources for future breeding programs.

## Significance

Upland cotton (*Gossypium hirsutum* L.) underpins the global cotton industry, yet the genetic mechanisms driving its domestication remain poorly resolved. Here, we integrate a large-scale pan-genome of 2,910 accessions to clarify *G. hirsutum*’s single domestication origin and delineate three previously uncharacterized key stages of its evolutionary domestication. We innovatively pinpoint two pivotal genes: *GhTOFD06*, a regulator of photoperiod sensitivity, and GhSID05, an asparaginase-encoding gene as a controller of seed yield. Notably, our analysis also uncovers natural gene flow between *G. hirsutum* and *G. barbadense*. These findings provide a high-resolution framework for cotton domestication and deliver actionable molecular targets to accelerate breeding for addressing critical needs of the global textile and agricultural sectors.

Crop domestication has played a crucial role in shaping human civilization (1, 2). Identifying the genetic variations behind this process can uncover the complex principles of agricultural evolution (3). At the genetic level, crop domestication typically involves multiple changes, including decreased photoperiod sensitivity, which is crucial for adapting to different environments (4). Other targeted traits include increased yield and quality, leading to improved food, feed, fiber, and fuel production and quality (5). Modifications in plant architecture, such as the arrangement of shoots, branches, leaves, and flowers arrangement, are also favored for easier management and to reduce damage from wind or heavy rains. Shortening growth cycles allows for faster harvests, while modifications in flower maturation and seed shattering or shedding are equally important (1). Understanding the genetic components of crop domestication can facilitate the development of improved crop varieties with enhanced yield, better quality, and greater resistance to environmental stress, and accelerate the domestication of wild relatives of crop species.

Upland cotton is a highly domesticated ecotype of *G. hirsutum*, cultivated globally for its high yield, broad adaptability, and excellent fiber quality, making it a crucial raw material for the textile industry (6). Archaeological evidence from the Tehuacán Valley of Mexico indicates *G. hirsutum* dates back 4,000 to 5,000 y (7), with its domestication history recently contextualized by a landmark graph pan-genome study of allotetraploid cotton (8). This study validated a three-stage domestication paradigm (wild → landrace → cultivar) and identified a dual diversity center in the Central America-Caribbean region, providing a valuable macroevolutionary framework for cotton domestication. However, constrained by the relatively limited number of reference genomes, this graph pan-genome study leaves unresolved the fine-scale genetic dynamics within each domestication stage—gaps that can be addressed by leveraging diverse wild and landrace resources. Multiple wild populations of *G. hirsutum* have been observed across Mesoamerica and the Caribbean, including races *yucatanense*, *punctatum*, *latifolium*, *richmondi*, *palmer*, *morrilli*, and *marie-galante* (9, 10). The Yucatan Peninsula in Mesoamerica is believed to be the original site where *G. hirsutum* was first domesticated, with *yucatanense* considered its most primitive form (11, 12). *Punctatum* overlaps with *yucatanense* in distribution and represents a sprawling perennial shrub of *G. hirsutum* (11). Subsequently, *punctatum* spread throughout Mesoamerica and the Caribbean, giving rise to *latifolium*, *richmondi*, *palmer*, and *morrilli* (11). Race *marie-galante*, found in the Caribbean, northern South America, and Central America, exhibits unique morphological characteristics, though details of its population formation are still unclear. These cotton landraces display prolonged growth periods, extreme photoperiod sensitivity, lower fiber yield and quality, and perennial features (11). Since the genetic variations behind upland cotton domestication traits are still not well understood, these wild accessions could help fill this knowledge gap and provide a broader range of genetic resources to improve our understanding of upland cotton domestication.

This study employed a comprehensive approach, integrating whole-genome resequencing, population evolutionary analysis, and genome-wide association analysis (GWAS), to elucidate the historical trajectory of upland cotton domestication. Our findings highlight key factors influencing the diversification of *G. hirsutum*, including domestication of fiber quality and yield-related traits, which facilitated its global dispersal. We identified pivotal genes involved in upland cotton domestication and demonstrated that the gradual reduction in photoperiod sensitivity was a critical trait during early cotton domestication and subsequent genetic improvements, which play a significant role in expanding upland cotton cultivation into higher-latitude regions.

## Results

### A Comprehensive Variation Map of *G. hirsutum*.

To capture the extensive genetic diversity of *G. hirsutum*, we analyzed a whole-genome resequencing dataset. This dataset contained 2,910 samples, of which 2,559 were *G. hirsutum*, spanning a broad range of wild and cultivated varieties; 323 were *G. barbadense,* and 28 were outgroup species (Dataset S1). Our sequencing efforts yielded 5,259,841 SNPs and 3,095,886 InDels, representing a significantly larger number of variants than previously reported (Dataset S2) (13, 14). Notably, 0.117% (7,546) of SNPs and 0.293% (12,390) of InDels were predicted to affect gene functions (13,711 genes) (Datasets S3 and S4), with significant enrichment in pathways like oxidation-reduction (GO:0055114), protein phosphorylation (GO:0006468), and pollen recognition (GO:0048544) (Dataset S5).

### The Genetic Diversity of the Predomestication *G. hirsutum* Population.

**Fig. 1.**
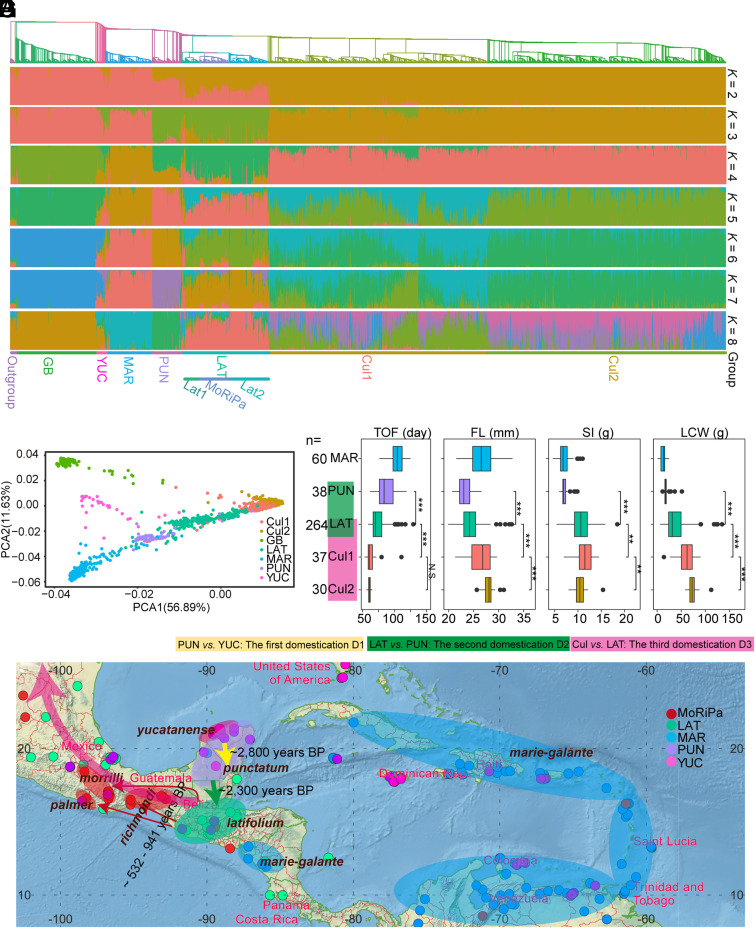
Phylogeny and population structure of *Gossypium* accessions. (*A*) Maximum likelihood phylogenetic tree and population structure of core cotton accessions. (*B*) Principal Component Analysis (PCA) showing PCA1 versus PCA2. (*C*) Geographic distributions of *G. hirsutum* races in Central America. Dots indicate different populations reconstructed from population genomics. The red arrow and time on the arrow indicate the direction and estimated spread time of the *G. hirsutum* accession, based on previous reports and population genomics inference. (*D*–*G*) Quantification of time to flowering (TOF), fiber length (FL), seed index (SI), and lint cotton weight (LCW) from five groups of *G. hirsutum*. ***P < 0.001, two-tailed *t* test.

We reconstructed phylogenetic relationships among 2,910 accessions using 36,028 high-quality SNPs at fourfold degenerate synonymous sites (4DTv). Phylogenetic analysis revealed that all contemporary cultivated *G. hirsutum* accessions formed a monophyletic clade, supporting a single domestication origin for modern cultivated upland cotton. The *G. hirsutum* races were situated at the base of the *G. hirsutum* branch and further divided into four distinct groups: *yucatanense* (YUC, n = 36), *marie-galante* (MAR, n = 190), *punctatum* (PUN, n = 120), and *latifolium* (LAT, n = 350), corresponding to the dominant racial components within each group. Group LAT can be further divided into three subgroups: MoRiPa (n = 73), Lat1 (n = 119), and Lat2 (n = 158) (Fig. 1*A*). These clustering results were supported by population structure analysis (Fig. 1*A*) and PCA clustering analysis (Fig. 1*B*).

The YUC group occupies a basal position (Fig. 1*A*), which exhibits significantly higher nucleotide diversity (π = 0.127 ± 1.14E^−04^) and a rapid decay of linkage disequilibrium (LD) compared to other groups and subgroups (*SI Appendix*, Fig. S1 *A* and *B*). This supports the idea that *yucatanense* is more closely related to the ancestral form of *G. hirsutum* than the other groups (*SI Appendix*, Fig. S1*A*). The MAR group has a widespread distribution across southern Central America, northern South America, and the Caribbean(7), which genetic components and phenotypic characteristics are clustered closer to *G. barbadense* (GB, n = 323). This confirms previous suggestions that natural hybridization occurred between *G. hirsutum* and *G. barbadense* prior to *G. hirsutum* domestication (Fig. 1*A*, *SI Appendix*, Fig. S1*C*, and Dataset S6). The PUN group, previously nominated as the earliest domesticated form of *G. hirsutum* (7), exhibits moderately lower genetic diversity (π = 1.339 × 10^−04^ ± 1.16E-04) (Fig. 1*A* and *SI Appendix*, Fig. S1*B*). Notably, several insular accessions collected from southern China (Hainan and Guangdong province), previously called *purpurascens*, belong to this group (Dataset S1). The LAT group is the closest clade to cultivated accessions (Cul1: n = 893; Cul2: n = 970), exhibiting significantly higher diversity in yield and fiber quality (Coefficient of variation, CV = 0.010 ~ 0.564; Shannon index = 5.435 ~ 5.759) (Dataset S6).

### The Origin of Modern Upland Cotton.

Using the sequentially Markovian coalescence (SMC++) method, we estimated the split time between the YUC group and PUN group to be approximately 2,800 y BP (Fig. 1*C* and *SI Appendix*, Fig. S2). This was followed by the emergence of the race *latifolium* in Mesoamerica around 2,300 y BP, which subsequently diversified into more races: *richmondi*, *palmeri*, *morrilli*, and a new race *latifolium* over the past millennium.

We estimated the effective population size (*N*e) of PUN, Lat2, and MoRiPa, and found that it is projected to decline rapidly toward extinction in the near future (*SI Appendix*, Fig. S2). This highlights the urgent need for conservation efforts to ensure the survival of these populations. To quantify the level of population differentiation, we calculated the paired fixation index (*F*_ST_) between groups and subgroups. Combined with GB, the results showed that the MAR and GB groups have the lowest *F*_ST_ (0.654) (*SI Appendix*, Fig. S1*D*), which is consistent with the genetic admixture of MAR and GB revealed by population structure analysis (Fig. 1*A*). Furthermore, we found that Lat1 exhibits the lowest *F*_ST_ (0.0836 and 0.1323) compared to Cul1 and Cul2, suggesting that the race *latifolium* in Lat1 may be the closest ancestor of most modern cultivated upland cotton (*SI Appendix*, Fig. S1*E*).

Our large-scale genomic analysis and the geographic distribution of races suggest that upland cotton domestication may have experienced three stages (Fig. 1*C* and *SI Appendix*, Fig. S1). The first stage (D1) involved the initial domestication of the wild progenitor *punctatum* in tropical Central and South America. The second stage (D2) focused on the utilization of *latifolium*. During this phase, attention was given to characteristics such as fiber length (FL), seed index (SI), and lint cotton weight (LCW) through selective empirical selection efforts to enhance these traits. Simultaneously, efforts were made to identify variants with shortened flowering time to increase agricultural productivity and adaptability to a wider range of growing conditions. In the third stage (D3), the primary focus was to further improve fiber quality (as observed by FL) and yield (as observed by LCW). Further shortening of flowering time enabled cotton to better adapt to modern agricultural conditions, including shorter growing seasons and increased mechanized harvesting demands (Fig. 1 *D*–*G* and *SI Appendix*, Figs. S3–S5).

### Genomic Signals of Upland Cotton Domestication.

**Fig. 2.**
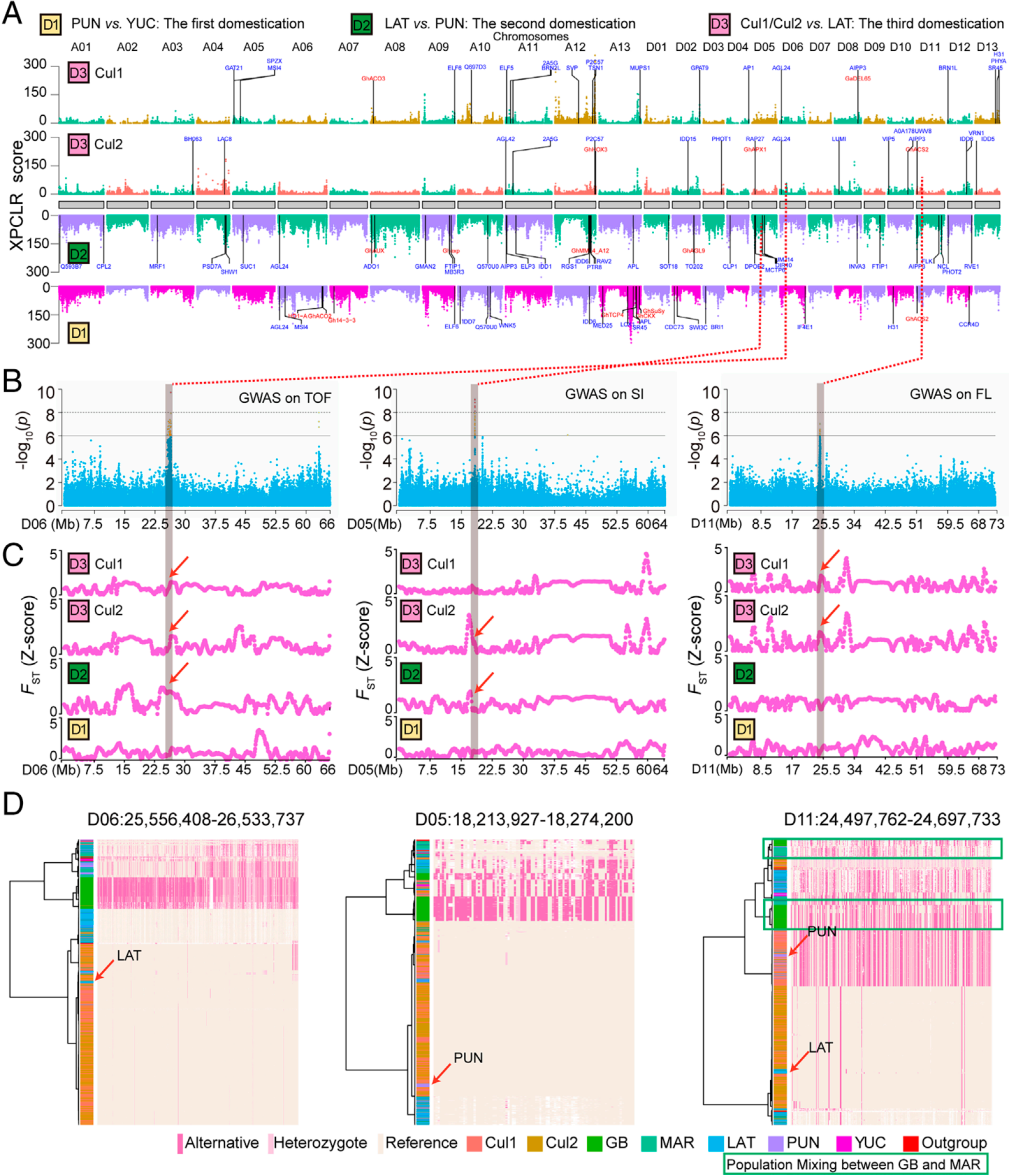
Genome-wide selective sweep analyses using the Ross-population composite likelihood ratio test across five groups (YUC, PUN, LAT, and Cul1/Cul2). (*A*) Genome-wide selective signals between Cul1/Cul2 versus LAT (third domestication, D3), LAT versus PUN (second domestication, D3), and PUN versus YUC (first domestication, D1), respectively. (*B*) Local Manhattan plots for three domestication traits obtained from GWAS signals. Black lines indicate the threshold for GWAS (–log_10_(p) < 6 and 8). TOF, time to flowering; FL, fiber length; SI, seed index. (*C*) highly divergent genomic regions (Z-score of *F*_ST_ > 2) and their overlap with GWAS signals. Red arrows indicate these divergent regions that overlap with GWAS signals shown in panel (*B*). (*D*) Heatmaps for haplotype of linkage Disequilibrium (LD) block from three GWAS signals. Each row is a cotton accession, and each column is a haplotype. The haplotypes relative to reference genome TM-1 were defined for each accession.

To investigate the impact of natural and artificial selection on population divergence during the domestication of upland cotton across three stages, we performed genome-wide selective sweep analyses using the Ross-population composite likelihood ratio test (XP-CLR) across five groups and subgroups (YUC, PUN, LAT, and Cul1/Cul2). Our analysis identified 1,369, 1,345, and 1,160/871 potential selective sweep loci between PUN versus YUC (D1 stage), LAT versus PUN (D2 stage), and Cul1/Cul2 versus LAT (D3 stage), respectively (Fig. 2*A* and Dataset S7). These loci collectively account for 2.8 to 4.5% of the genome. We also confirmed several selective sweep regions overlapping with XP-CLR findings through *F*_ST_ analysis and decorrelated composite of multiple signals (DCMS) (15) (*SI Appendix*, Figs. S6–S11).

A total of 12,328 genes are located within selective regions, with 22 genes identified across all three domestication events in upland cotton, indicating prolonged and consistent selection during domestication (*SI Appendix*, Fig. S12 and Dataset S8). Among these genes, *Gh_A13G20800* encodes the EIN3-binding F-box protein 1 (EBF1), a component of the SCF (SKP1-cullin-F-box) E3 ubiquitin ligase complex. SCF complexes are known to regulate the ethylene signaling cascade, which is crucial for controlling fiber initiation and elongation, thereby influencing fiber yield and quality (16, 17). Additionally, EBF1 has been implicated in promoting elongated fruit shape in tomato (18). Another gene, *Gh_A12G136200*, encodes a pectin acetyl esterase (PAE), which hydrolyzes acetyl esters in the homogalacturonan regions of pectin in the primary cell wall (19). Pectin modifications in the primary cell wall are critical for fiber elongation (16).

Through selective scanning analysis, 15 candidate genes related to yield and quality were identified (Fig. 2*A* and Dataset S9). Multiple genes involved in regulating fiber initiation and elongation networks across the three domestication stages were found. These include several genes within the ethylene-regulated fiber elongation signaling pathway, such as *GhACS*2 (*Gh_D11G024300*), *GhACO2-3* (*Gh_A06G179300* and *Gh_A08G059100*), *GhDEL65* (*Gh_D08G203800*), *GhSusy* (*Gh_A13G215000*), *GhAPX1* (*Gh_D05G098900*), *GhEXP* (*Gh_A09G175200*), and *GhHD1* (*Gh_A06G172400*), indicating sustained selection for fiber yield and quality (*SI Appendix*, Fig. S13*A*). Additionally, we identified 79 known candidate genes already associated with flowering time (Fig. 2*A* and Dataset S10). Among these, genes encoding photoperiod-regulating factors such as *SOC1* (*Gh_A11G007600*), *PHYA* (*Gh_D13G199100*), *SVP* (*Gh_D06G033700*), and *AP1* (*Gh_D13G199100*) were selectively favored during the domestication of modern cultivated upland cotton (*SI Appendix*, Fig. S13*B*). Moreover, we found that genes encoding the flowering-time control protein (FCA, *Gh_D10G197200*) and the ethylene-responsive transcription factor (RAP27, *Gh_D05G098700*), which facilitate the transition from the vegetative meristem to reproductive development, were also subject to selection. These results suggest that the global expansion of modern cultivated upland cotton is driven not only by human selection for yield and quality but also by changes in photoperiod sensitivity.

Several selective sweep loci identified by XPCLR show notable overlaps with signals from genome-wide association studies (GWAS), including loci associated with time of flowering (TOF), SI, and FL (Fig. 2*B*). Specifically, the TOF-associated locus, *GhTOFD06*, is located in the genomic region D06:25,556,408-26,533,737. This region overlaps with selective sweep signals observed during both the D2 and D3 stages, as supported by fixation index (*F*_ST_) (Fig. 2*C*). Additionally, the haplotypes of Cul1/Cul2 align with LAT in this region (Fig. 2*D*), which may help explain the gradual replacement of upland cotton by the race *latifolium*, introduced from the Mexican highlands to North America in the 19th century (7). The FL association locus, *GhFLD11*, is supported by selective sweep signals (*F*_ST_) observed during the D3 stage (Fig. 2*C*). The MAR group can be categorized into two haplotypes, each derived from different GB groups, suggesting that genetic admixture between MAR and GB groups may drive potential innovations in fiber traits (Fig. 2*D*). Additionally, the Cul2 haplotype is derived from the PUN group, while the Cul1 haplotype originates from the LAT group. Considering the single domestication origin of cultivated upland cotton, this admixture may be related to the introgression of gene fragments from other *G. hirsutum* races. The SI association locus, *GhSID05*, is supported by selective sweep signals observed during the D2 stage (Fig. 2*C*). The haplotype in upland cotton cultivars is derived from the PUN group, with relaxed selection in modern upland cotton breeding (Fig. 2*D*).

### Domestication of Flowering in Modern Upland Cotton.

**Fig. 3.**
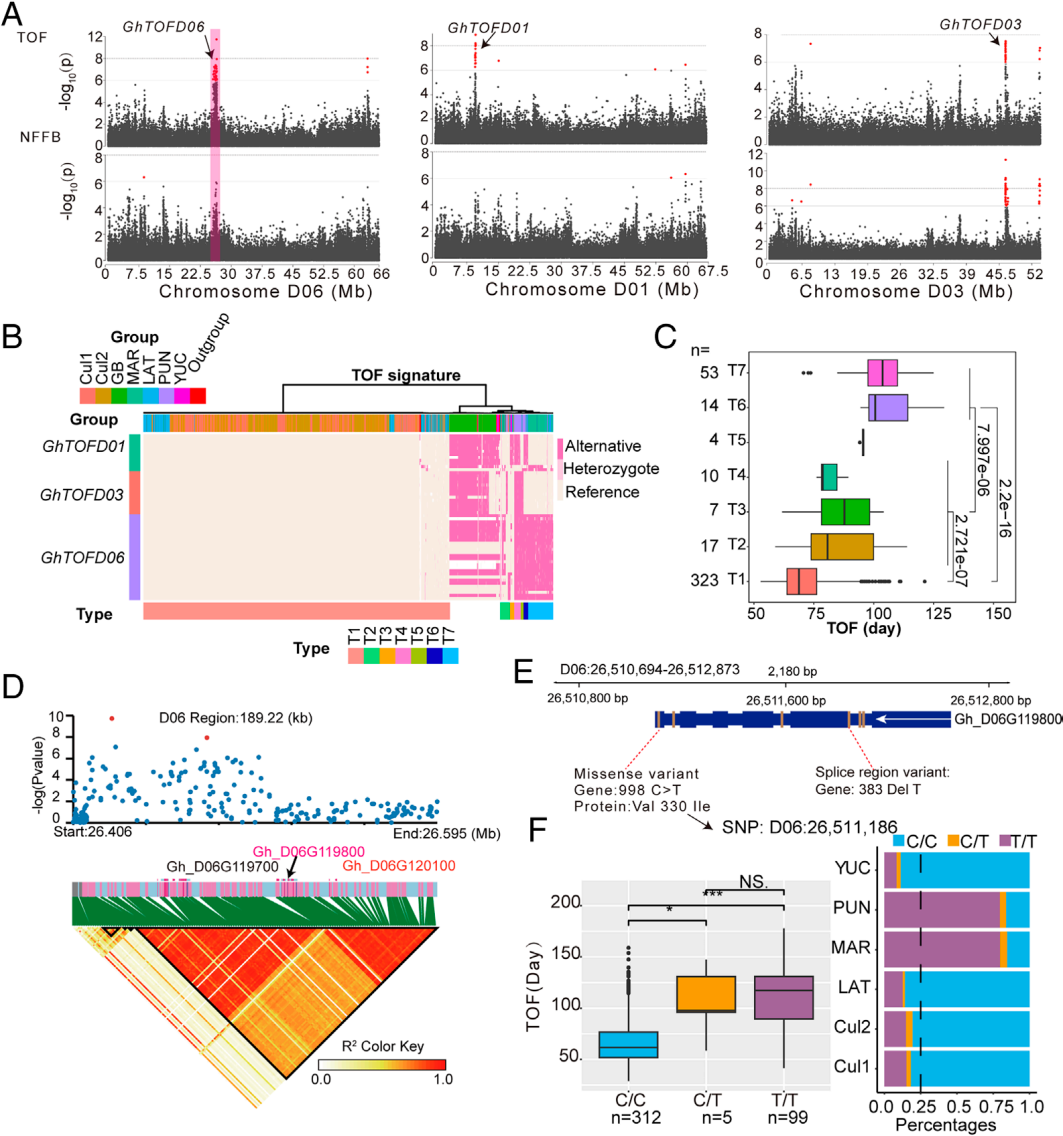
GWAS for time to flower (TOF) and the node of the first fruiting branch (NFFB), and identification of the candidate gene *GhTOFD06* and *GhTOFD01*. (*A*) Manhattan showed the TOF and NFFB based on GWAS. significance threshold (−log_10_(P)>6 and >8, Bonferroni correction). (*B*) Heatmap displays the time to flowering (TOF) signature in *GhTOFD01*, *GhTOFD03*, and *GhTOFD06* relative to the reference genome TM-1. Accessions (columns) are grouped according to the categories shown in Fig. 1 (colored bars). Using the ward.D clustering method, the TOF signature genotypes of *GhTOFD01*, *GhTOFD03*, and *GhTOFD06* were classified into seven types. (*C*) The quantification of TOF of seven types. Statistical hypothesis test: two-tailed *t* test. (*D*) Local Manhattan plot (*Top*), gene distribution (*Middle*), and local LD heatmap (*Bottom*) around the peak associated with *GhTOFD06*. (*E*) Gene structure and the variation site of *GhTOFD06.* (*F*) Boxplots for TOF based on a variation in D06:26,511,186 (*Left*) (***P < 0.0001, two-tailed *t* test in a pairwise comparison), and Frequency changes of D06:26,511,186 in different *G. hirsutum* populations. Center line, 25% (*Right*).

Reduced sensitivity to photoperiod, which enables normal flowering in long daylight regions, is a critical step in domestication that facilitated the global spread of *G. hirsutum* from tropical areas. As expected, flowering time became progressively earlier during the second and third domestication stages of upland cotton (Fig. 1*C*). Through GWAS, we identified three TOF loci on chromosomes D01, D03, and D06 (Fig. 3*A*). Notably, the loci *GhTOFD03* and *GhTOFD06* overlapped with those linked to the first fruiting branch node (NFFB), an important trait determining maturity in cotton. Although the *GhTOFD03* locus is consistent with previous studies (20, 21), we did not detect any overlapping selective sweep signals associated with it.

Genotypes at the TOF loci *GhTOFD01*, *GhTOFD03*, and *GhTOFD06* can be classified into seven distinct haplotypes (T1-T7) (Fig. 3*B*). Notably, most modern cultivated upland cottons belong to haplotype T1, which has the shortest flowering time. Haplotype T2 to T4 exhibit significantly longer flowering times than T1, while haplotypes T6 and T7 have the latest flowering times compared to the others (Fig. 3*C*). A candidate gene for *GhTOFD06*, *GhD06G119800*, encodes the COP9 signalosome complex subunit 5b (CSN5B), a homolog in *A. thaliana* that is involved in photomorphogenesis (Fig. 3*D*) (22, 23). A nonsynonymous mutation at D06:26,511,186 (C > T) was found to correlate with delayed flowering in *G. hirsutum* (Fig. 3*E*). The homozygous wild-type genotype (C/C), associated with a short TOF was predominantly present in the LAT, Cul1, and Cul2 populations (Fig. 3*F*). However, the YUC group, which also has more extended growth periods, exhibits a recessive mutation genotype at D06:26,511,186. *GhTOFD06* was expressed at significantly higher levels in the anthesis buds of accession TX2094 (YUC), which shows a long TOF, compared to the modern cultivar TM-1 (Dataset S11). This pattern supports its potential role as a negative regulator of flowering time, as previously observed in tomato (24).

We further analyzed the haplotype diversity of *GhD06G119800* across 2,910 cotton accessions, identifying seven genotypes (*SI Appendix*, Fig. S14*A*). Despite possessing the early-flowering mutation D06:26,511,186, the YUC group mainly comprises TOF2 haplotypes with additional SNP variations beyond D06:26,511,186, distinguishing it from TOF5 haplotypes associated with early flowering. In contrast, the PUN and MAR groups, known for longer flowering periods, predominantly carry the TOF10 haplotype. Among cultivated varieties, TOF5 and TOF8 haplotypes are prevalent, with TOF8 linked to earlier cotton cultivars and TOF5 primarily originating from the LAT group (*SI Appendix*, Fig. S14*B*). Our findings suggest that the domestication of upland cotton’s flowering time primarily occurred during the third domestication stage (D3). Genotypes carrying the dominant gene D06:26,511,186 (TOF9/10) exhibit longer flowering times (*SI Appendix*, Fig. S14*C*). Haplotypes TOF9/10, associated with extended flowering periods, are predominantly found in low latitude regions, while TOF5 haplotypes with shorter flowering periods are globally distributed (*SI Appendix*, Fig. S14*D*).

### Domestication of Quality-Related Traits.

**Fig. 4.**
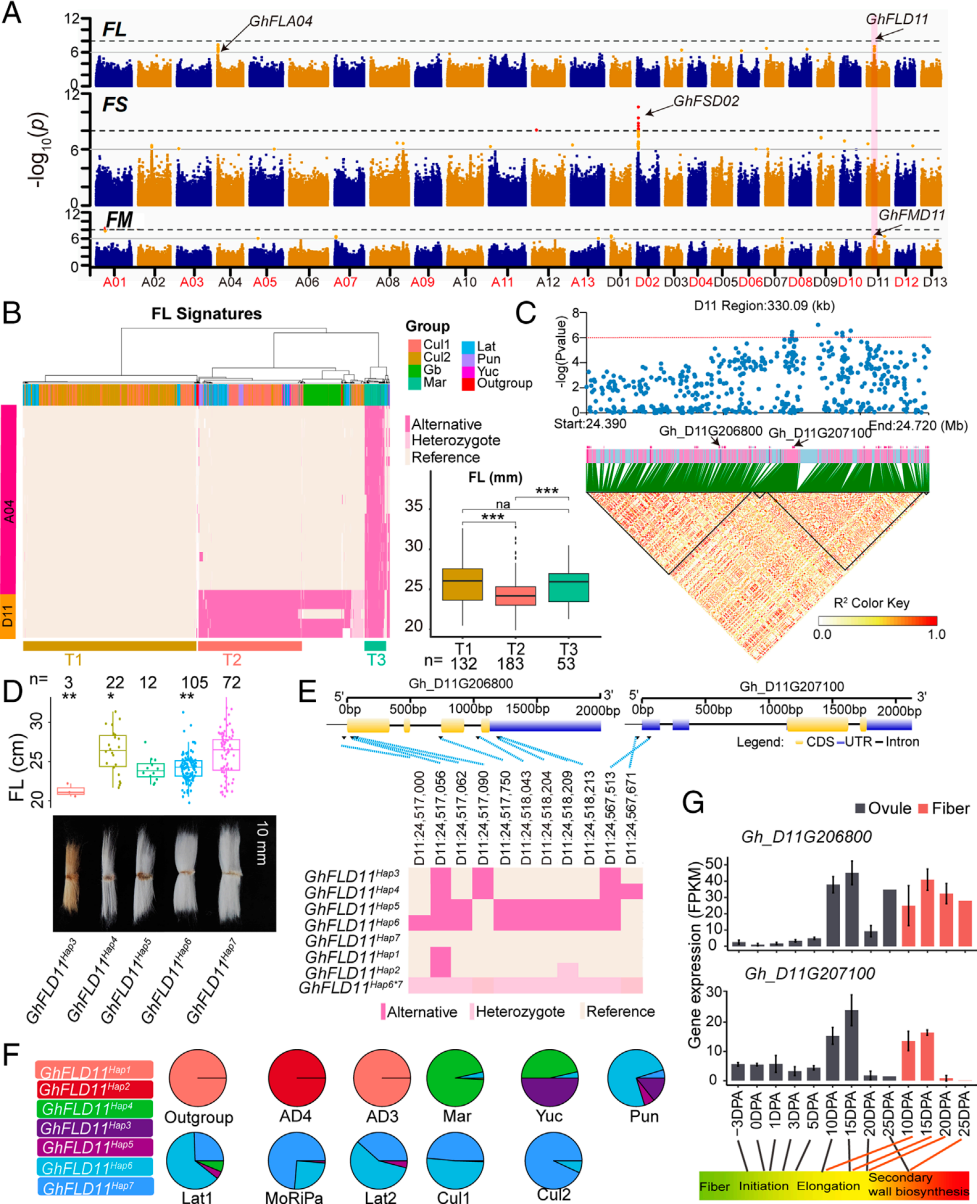
GWAS for fiber length (FL) and identification of the candidate gene *GhFLD11*. (*A*) Manhattan showed the fiber traits based on GWAS. The horizontal dashed line represents the significance threshold (*P* < 1 × 10^−6^ and *P* < 1 × 10^−8^, Bonferroni correction). (*B*) Heatmap displays the fiber length (FL) signature in *GhFLA04* and *GhFLD11* relative to the reference genome TM-1. Accessions (columns) are grouped according to the categories shown in Fig. 1 (colored bars). Using the ward.D clustering method, the FL signature genotypes were classified into three types of *G. hirsutum* (T1, T2, and T3). The quantification of fiber length (FL) of three types. Statistical hypothesis test: two-tailed *t* test. ***P < 0.0001. (*C*) Local Manhattan plot (*Top*), gene distribution (*Middle*), and local LD heatmap (*Bottom*) around the peak associated with FL. (*D*) Phenotypic features of fiber from five haplotypes of *G. hirsutum*. (Scale bar: 10 mm.) (*E*) Gene structure and the variation site of *GhFLD11* (*Top*), the major different haplotypes of *GhFLD11* (*Bottom Left*), and the distribution of FL in different haplotypes (*Bottom Right*). (*F*) The distribution of haplotypes in different *G. hirsutum* population (pie plot). Center line, median; *(*P* < 0.01, two-tailed *t* test). (*G*) RNA-seq analysis of the expression of *Gh_D11G206800* and *Gh_D11G207100* in ovule and fiber during fiber initiation, elongation, and secondary wall biosynthesis. Error bars, mean ± SD.

Cotton FL, fiber strength (FS), and fiber micronaire value (FM) are crucial indicators of textile properties and represent key traits targeted in cotton domestication and breeding programs. Through GWAS, we identified two fiber length-associated loci on A04 and D11, along with a fiber strength-associated locus on D02 (Fig. 4*A*). In the entire *G. hirsutum* population, the combined genotypes of *GhFLA04* and *GhFLD11* loci could categorize the accessions into three distinct haplotypes (T1-T3) (Fig. 4*B*). The majority of haplotype T1, predominantly composed of the Cul2 subgroup, exhibits longer fiber length (Fig. 4*B*). However, haplotype T3, which includes accessions from the MAR subgroup, also displays longer fiber length. These results suggest that the MAR subgroup of the *GhFLA04* locus contains untapped genetic resources for improving upland cotton fiber length.

We noticed that the FL locus, *GhFLD11*, overlaps with the fiber micronaire value (FM) locus *GhFMD11* (Fig. 4*A*). This genomic region encompasses 19 candidate genes. *GhKPR6* (*Gh_D11G206800*) was previously identified as a candidate gene for *GhFLD11* (*FL2*) (13, 20). This gene is directly regulated by *GhBES1.4* (*BRI1-EMS-SUPPRESSOR1*), which influences brassinosteroid (BR)-mediated regulation of cotton fiber cell elongation (Fig. 4*C*) (25). We found nine SNPs within *GhKPR6*, including D11:24,517,062 (G->T), which has previously been linked to upland cotton fiber length (18). In this study, a nonsynonymous mutation, D11:24517090 (C:73G>T, Pro: Ala25Ser), was identified and appears significantly correlate with fiber length (Fig. 4*D*).

Interestingly, *BRI1 KINASE INHIBITOR 1* (*GhBKI1*), another candidate gene within the genomic region of *GhFLD11* loci, is involved in negatively regulating BR (Fig. 4*C*) (26). Two SNPs were found to be significantly associated with fiber length, one of which was a 5′ UTR premature start codon gain variant specifically carried by accessions from the MAR group (Fig. 4*E*). By integrating the analysis of both genes, we found that their genotypes could be categorized into seven haplotypes within the entire *G. hirsutum* population (Fig. 4*E*). Among cultivated accessions, GhFLD11^Hap6^ and GhFLD11^Hap7^ are prominent, with the latter showing a gradual increase in frequency during upland cotton domestication, highlighting a pronounced selection preference (Fig. 4*F*). Furthermore, *GhBKI1* exhibits increased expression during the fiber elongation stage in cultivated cotton varieties (Fig. 4*G*), suggesting its potential role, along with *KPR6*, in regulating BR signaling and contributing to fiber elongation.

### Domestication of Yield-Related Traits.

**Fig. 5.**
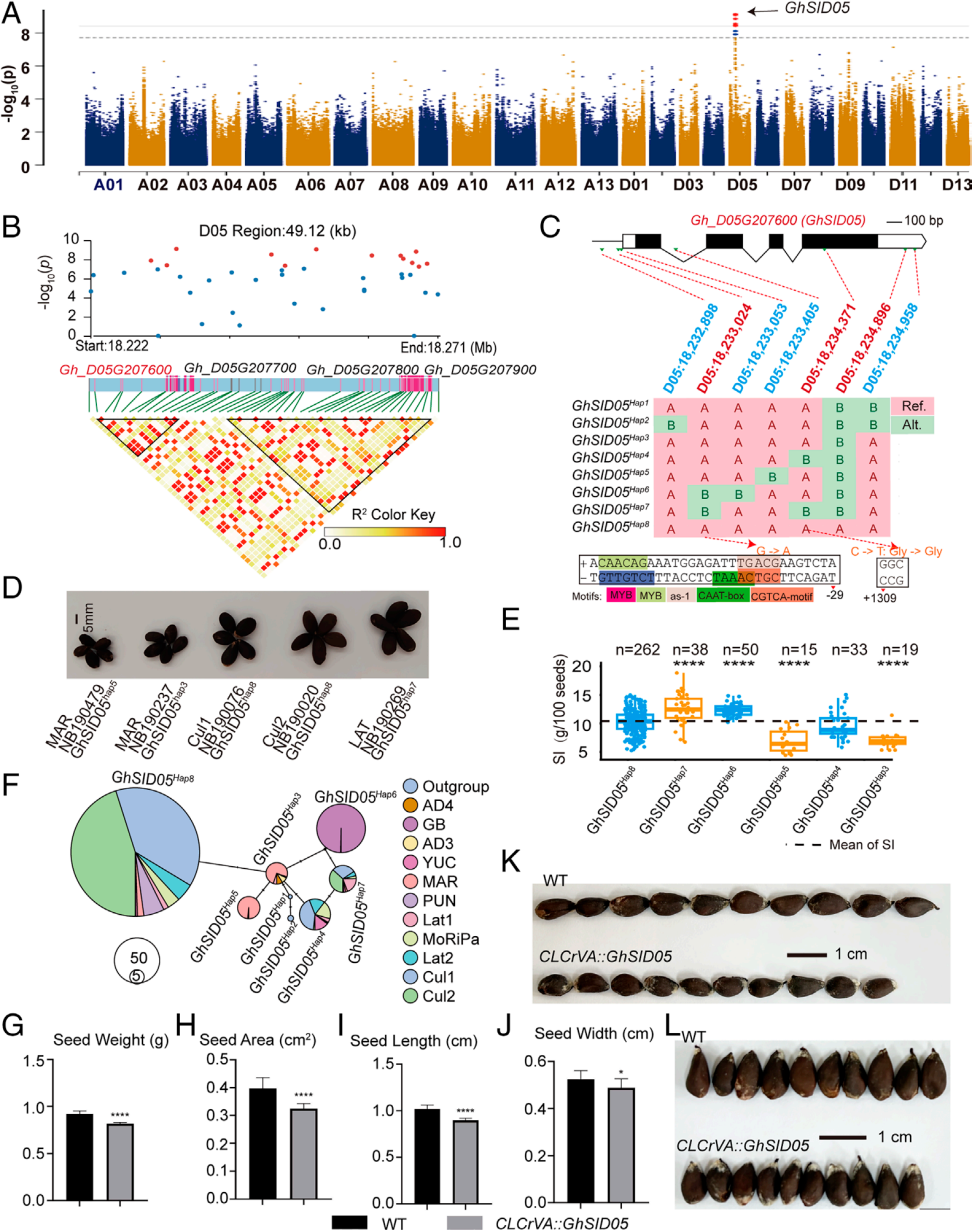
GWAS for seed index (SI) and functional identification of the candidate gene *GhSID05*. (*A*) Manhattan showed the SI based on GWAS. significance threshold (−log10(P) > 7.8 and >8.2, Bonferroni correction). (*B*) Local Manhattan plot (*Top*), gene distribution (*Middle*), and local LD heatmap (*Bottom*) around the peak associated with SI. (*C*) Gene structure and the variation site of *GhSID05* (*Top*), the major different haplotypes of *GhSID05* (*Middle*), and the cis-regulatory elements in *GhSID05* promoter. (*D*) Phenotypic features of seed in cotton accessions. (Scale bar: 5 mm.) (*E*) The distribution of SI in *G. hirsutum* accessions with six different haplotypes. Center line, median; ***(*P* < 0.0001, two-tailed *t* test). (*F*) Haplotype network of eight haplotypes in 2,910 cotton accessions. (*G*–*J*) Statistical analysis of seed size and weight in WT versus Silenced lines: (*G*) Seed weight (*G*), (*H*) Seed area (cm^2^), (*I*) Seed width (mm), (*J*) Seed length (mm). Error bars represent ± SD (n = 3 biological replicates, each with 10 seeds). Statistical significance determined by two-tailed Student’s *t* test: *P < 0.05, **P < 0.01, ***P < 0.001. (*K* and *L*) The phenotypes of seed length (*K*) and width (*L*) comparison between wild-type (WT) and GhSID05-silenced (Silenced) CRI49 lines.

For yield-related traits, we identified five overlapping loci associated with lint cotton weight (LCW), seed cotton weight (SCW), and boll weight (BW) on chromosomes A03, A13, D01, and D13, respectively. Additionally, a locus overlapping SCW, BW, and SI was found on chromosome D05 (Fig. 5*A* and *SI Appendix*, Fig. S15). Among the candidate genes at this locus, *GhSID05* (*Gh_D05G207600*), which encodes an asparaginase, emerged as a candidate for cotton yield regulation (Fig. 5*B*).

Genotype analysis identified seven variants in *GhSID05* that formed eight haplotypes. Notably, the variant D05:18,233,024 is in a cis-acting regulatory element involved in MeJA responsiveness (Fig. 5*C*). Among these haplotypes, *GhSID05^Hap3-5^* exhibited a lower SI compared to *GhSID05^Hap6-8^* (Fig. 5 *D* and *E*). Multiple haplotypes were shared among *G. hirsutum* populations, possibly due to genetic flow between different populations after separation or relaxed selection on ancestral polymorphisms. *GhSID05* is situated within the selection interval of the second domestication of *G. hirsutum* (LAT versus PUN) (Fig. 5*F* and Dataset S7), with LAT, Cul1, and Cul2 all exhibiting higher SI. Therefore, continuous genetic flow among different populations is likely the primary reason for the shared haplotypes of *GhSID05* among *G. hirsutum* populations.

### Silencing of *GhSID05* Reduces Seed Size and Weight.

To experimentally validate the regulatory role of *GhSID05* in SI (a core yield-related trait), we performed virus-induced gene silencing (VIGS) using the CLCrVA vector in *G. hirsutum* cv. CRI49, a modern cultivar carrying the high-SI haplotype GhSID05^Hap8^. Reverse-transcription quantitative polymerase chain reaction (RT-qPCR) confirmed efficient gene silencing: GhSID05 expression was reduced by 24 to 64% in *CLCrVA:GhSID05* plants relative to wild-type (WT) controls (*SI Appendix*, Fig. S16). Phenotypic analysis of mature seeds revealed significant changes in SI-related traits: CRI49 silenced plants exhibited 11.41% lower single seed weight (*P-*value < 0.01), 11.8% shorter seed length (*P-*value < 0.01), 18.32% smaller seed area (*P-*value < 0.01), and 7.01% narrower seed width (*P-*value < 0.01) compared to the WT (Fig. 5 *G*–*K*). Our findings demonstrate that *GhSID05* is a key regulator of cotton SI, and its domestication-driven haplotype divergence contributes to seed trait improvement in cultivated cotton. This gene provides a promising target for molecular breeding to enhance cotton yield.

### Natural Introgression of *G. barbadense* Influences the Morphology of *G. hirsutum* Race *Marie-Galante*.

**Fig. 6.**
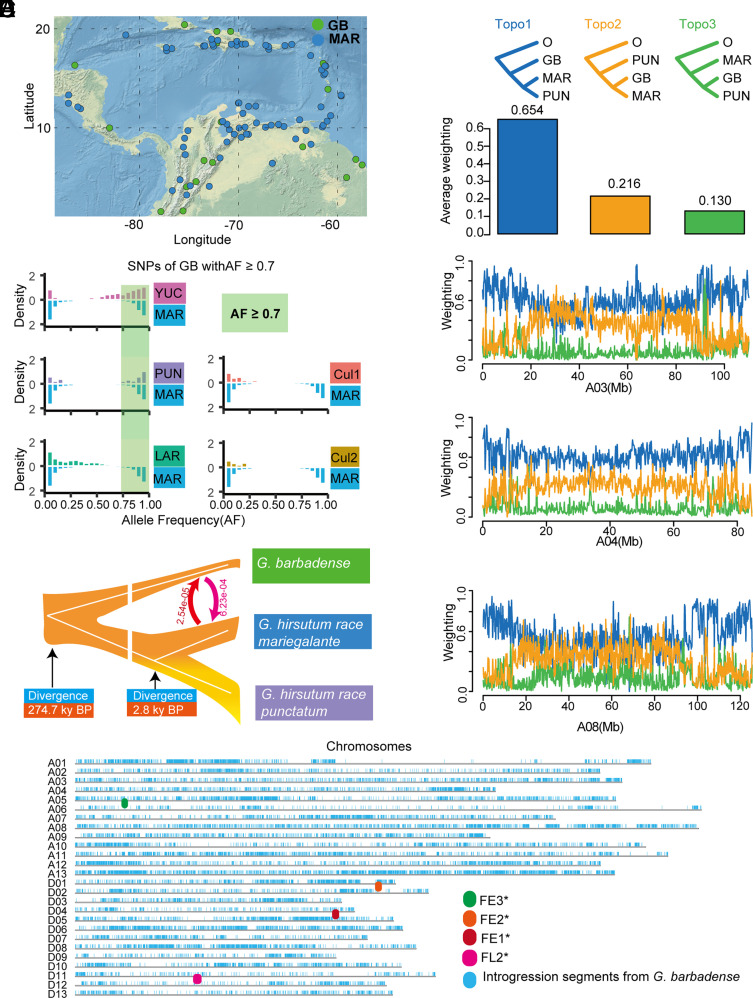
The evidence for the natural gene flow between *G. hirsutum* and *G. barbadense*. (*A*) Geographic distributions of *G. hirsutum* race *marie-galante* and *G. barbadense* in Central America. Dots indicate different populations reconstructed from population genomics. (*B*) The frequency distribution of alternative variant sites in *G. hirsutum* from different groups, where alternative allele frequencies exceed 0.7 in *G. barbadense*. (*C*) Average weights assigned to the three potential topologies [Outgroup (O), *G. barbadense* (GB), *G. hirsutum race marie-galante* (MAR), and *G. hirsutum* race *punctatum* (PUN)] across entire genomes. (*D*) Distribution of these average weights for all three topologies described in (*C*) across chromosomes A03, A04, and A08, where the average weighting exceeds 0.3, using sliding windows. (*E*) Simplified schematic of the demographic scenario modeled by fastsimcoal29. Red and pink arrow indicates asymmetric gene flow. Numbers in brackets indicate 95% CI of estimated times. (*F*) The whole-genome distribution of introgressed segments in the *G. hirsutum* race *marie-galante* identified using the ABBABABA test. *ref. 13.

It has been previously reported that *G. barbadense* was first domesticated in the intermontane regions of the Northwest Andes and subsequently diverged into distinct populations due to the Andean range (14). Notably, the geographical distribution of *G. barbadense* landraces in the northern Andes and the Caribbean overlaps with that of the MAR group (Fig. 6*A*), providing opportunities for early interspecific introgression between *G. hirsutum* and *G. barbadense* accessions.

Based on the *G. hirsutum* reference genome (acc. TM-1), a substantial number of sites were missing in GB (missing rate > 0.8; 164,543 sites) (Dataset S12). Although high-frequency SNPs from GB are rarely observed in *G. hirsutum* groups, such as LAT, Cul1, and Cul2, some of these SNPs (allele frequency > 0.7) are notably common in the MAR group (Fig. 6*B* and *SI Appendix*, Fig. S17). We used the topology weighting by iterative sampling of subtrees (Twisst) method to examine the phylogenetic relationships among PUN, MAR, GB, and the outgroup. Our findings showed that most topology structures (65.4%) support the relationship ((PUN, MAR), GB, Outgroup), while 21.6% favor the topology ((GB, MAR), PUN, Outgroup) (Fig. 6*D* and Dataset S13). Notably, this proportion exceeds 30% on chromosomes A03, A04, and A08 (Fig. 6*E* and *SI Appendix*, Fig. S18).

We then used fastsimcoal (27) to evaluate nine models focusing on the origin of the MAR population and gene flow among the PUN, GB, and MAR populations (*SI Appendix*, Fig. S19). The optimal model supported our hypothesis, suggesting that after the divergence of race *marie-galante* and race *punctatum* approximately 2,800 y ago, there has been ongoing asymmetric bidirectional gene flow with *G. barbadense* (Fig. 6*E*). To further confirm the genetic admixture resulting from historical hybridization events, we employed ABBA-BABA analysis to dissect MAR segments originating from GB. We found GB introgression segments spanning approximately 18.16% (~404.37 Mb) of the entire genome (Dataset S14). We observed that several reported fiber quality-related loci, including *GhTOFD11*/*FL2*, *FE1*, *FE2*, and *FE*(13), were located within these introgression segments, suggesting that they may have originated from GB or MAR (Fig. 6*F*).

Gene flow between the MAR and GB has resulted in a unique blend of phenotypic traits from both *G. hirsutum* and *G. barbadense*. Specifically, the race *marie-galante* exhibited palmate leaves similar to those of *G. barbadense* (*SI Appendix*, Fig. S20*A*). A GWAS identified a leaf morphogenesis locus (*GhLM*) located on chromosome D01 (*SI Appendix*, Fig. S20*B*), which aligns with the previously characterized *GhOkra* gene mapped in tetraploid cotton (*SI Appendix*, Fig. S20 *C* and *D*) (28, 29). Analysis of 2,910 *Gossypium* accessions revealed five variant sites in these genes that collectively form seven unique haplotypes (*GhLM*^hap1-7^) (*SI Appendix*, Fig. S20*E*). The formation of palmate leaves in *G. barbadense*, similar to those found in race *marie-galante*, is likely attributed to the restoration of the okra gene function caused by the insertion of G-C alternation at the D01:62,571,907 locus (*GhLM*^hap5^) (*SI Appendix*, Fig. S20*F*).

Stem trichome morphology varied across the cotton genus, with most *G. barbadense* accessions displaying a smooth stem and most *G. hirsutum* accessions displaying thickly pubescent stems, particularly in race groups PUN, LAT, and YUC (*SI Appendix*, Fig. S21*A*). A GWAS conducted on the stem trichome identified an associated locus (A06:113.600–113.698 Mb) that harbored the gene *GhSTRA06* (*Gh_A06G172400*). This gene encoding a homeobox-leucine zipper protein has previously been recognized as a key regulator of epidermal hair initiation (*SI Appendix*, Fig. S21 *B*–*D*) (30–33). Across the 2,910 cotton accessions, this gene contained 11 SNPs and two InDels, resulting in eight haplotypes (*SI Appendix*, Fig. S21*E*). Notably, *GhSTR*^Hap7^ and *GhSTR*^Hap8^ exhibited the densest stem trichome, predominantly in upland cotton and its wild lineages, including race *marie-galante*. In contrast, *G. barbadense* haplotypes *GhSTR*^Hap4^ and *GhSTR*^Hap5^ exhibited lower stem trichome density (*SI Appendix*, Fig. S21*F*). Haplotype network analysis demonstrated that *GhSTR*^Hap4^ and *GhSTR*^Hap5^ in *G. barbadense*, likely originated from *G. mustelinum* ((AD)_4_), while the haplotypes of *G. hirsutum*, including those of MAR, were derived from *G. tomentosum* ((AD)_3_), consistent with the established phylogenetic relationships within tetraploid cotton species (*SI Appendix*, Fig. S21*G*).

## Discussion

Building a comprehensive genetic map of *G. hirsutum* is essential for genetic improvement and germplasm resource conservation. While pan-genome construction strategies, predominantly relying on whole-genome assemblies from a limited number of samples, have been extensively developed to capture more structural variations, and cotton-specific pan-genomes have also been successfully established, the discovery of novel genetic diversity remains constrained by the narrow breadth and limited scope of sample coverage (8, 34–38). Additionally, previous studies have used numerous *G. hirsutum* cultivars to explore genetic diversity and breeding advances (13, 14, 20, 39–41). However, the limited genetic diversity within upland cotton populations restricts the development of breakthrough new varieties in breeding programs. Notably, *G. hirsutum* comprises seven recognized landraces that exhibit diverse morphological variations (7, 42, 43). Understanding the genomic basis of this diversity could reveal valuable loci with unique variations that could enhance the gene pool of modern cultivars. Our study confirmed the rich genetic diversity among these landraces and provided insights into the timing of divergence and domestication trajectory of *G. hirsutum* in Mesoamerica. Molecular evidence suggests that race *latifolium* is the source of modern cultivated cotton varieties, establishing a genetic framework for domestication. The genetic bottleneck in modern cultivars stems from both their single domestication event and intensive artificial selection, as well as environmental pressures.

Our findings indicate that upland cotton, similar to maize (44–46), common beans (47), and squash (48), was initially domesticated on the Yucatán Peninsula in Mexico (49). Evidence from domestication migration patterns suggests an association with the “milpa” cropping system during early agricultural development, which was crucial for the Maya civilization (49). We categorized the domestication of upland cotton into three stages based on population genetics and archaeological evidence: the first stage (D1), around 2,800 y BP, resulted in the emergence of the earliest domesticated form, race *punctatum*, from *yucatanense*. Approximately 2,600 y BP, a second domestication event (D2) led to the establishment of race *latifolium* as cultivation areas expanded. Approximately six centuries ago, during the Age of Discovery, earlier domesticated cotton cultivars were introduced to North America, potentially even to China (*purpurascens*). However, from 1806 onward (~150 to 200 y ago), cultivars derived from *latifolium* accessions collected in the Mexican highlands began to replace them in part (7, 12, 50). This replacement had a significant impact on shaping the modern gene pool of cultivated upland cotton. Notably, the ~18.16% genome-wide introgression from *G. barbadense* (predominantly enriching the MAR group) represents postdomestication genetic supplementation that does not alter the core single-origin domestication trajectory.

Flowering, fiber, and yield-related traits exhibit continuous gradient changes during upland cotton domestication, representing typical characteristics of the domestication syndrome; the significant association signals detected by GWAS, combined with the directional shifts in genotype frequencies throughout the domestication process, collectively confirm the close correlation of these traits with the domestication of upland cotton. Photoperiod insensitivity represents a hallmark of crop domestication. In upland cotton, reduced photoperiod responsiveness enabled tropical/subtropical cultivars to flower and fruit at higher latitudes during their global dissemination (43). During the third domestication stage (D3), selective pressures favored genes that control photoperiod-regulated flowering, accelerating adaptive expansion. The CO-FT regulatory module mediates floral induction through photoreceptor-stabilized CONSTANS (CO) transcription factor, activating *FLOWERING LOCUS T* (*FT*) expression (51). Here, we identified *GhTOFD06*, a homolog of Arabidopsis *COP9* signalosome complex subunit 5b (*CSN5B*), as a key regulator of flowering time in cotton. *CSN5B* modulates the activity of COP1-containing ubiquitin ligase complexes, which suppress photomorphogenesis in darkness (52). In plants, *cryptochrome 2* (*CRY2*) inhibits the formation of the COP1/SPA1 complex under blue light, stabilizing the CO protein and promoting *FT* transcription, thereby regulating photoperiodic flowering (53, 54). Notably, the tomato (*Solanum lycopersicum*) *FANTASTIC FOUR 1/2c* (*FAF1/2c*) locus interacts with *CSN5B* to modulate early flowering (24), suggesting a conserved role for CSN5B homologs in flowering regulation across species. For loci like *GhTOFD03* that show robust GWAS associations but lack corresponding selective sweep signals in our research, their relevance to cotton improvement (rather than core domestication) reflects two key scenarios: either their elite alleles were selectively enriched in specific breeding subgroups (e.g., early-maturing cultivars) rather than fixed across all domestication stages, or they are subject to polygenic selection that is not fully captured by XP-CLR, which are more sensitive to strong, genome-wide selective pressures. Our findings imply that *GhTOFD06* may act as a pivotal domestication locus facilitating upland cotton’s latitudinal expansion. Although further molecular evidence is required to confirm the precise mechanism by which *GhTOFD06* influences flowering time, this locus provides a genetic target for understanding the evolutionary trajectory of cotton domestication.

Brassinosteroids (BR) regulate the synthesis of very-long-chain fatty acids (VLCFA) in cotton fibers, promoting fiber elongation (55–58). *GhKPR6* (*Gh_D11G206800*), located on D11 and directly regulated by *GhBES1.4* (*BRI1-EMS-SUPPRESSOR1*), is identified as a candidate gene for fiber length-related loci (*FL2*) (20, 25). We identified a negative regulator of BR, *BRI1* (*Gh_D11G207100*), that influences fiber elongation by modulating expression. The heterozygous genotype at this locus exhibits superior fiber length and was selected during the domestication of upland cotton, offering insights for improving fiber length in cultivated varieties.

Cotton yield is predominantly determined by lint yield, its primary economic value driver (59). Notably, cotton seeds, an underutilized component, serve as versatile feedstocks for the food, cosmetics, pharmaceutical, and animal feed industries, offering significant potential to boost cotton’s comprehensive economic output (60). Our findings show that during *G. hirsutum* domestication, lint yield was substantially enhanced via artificial selection, while cottonseed-related traits were largely overlooked in modern cultivated varieties. Seed size is intrinsically linked to seed nutrient reserves and metabolism (61). As a key enzyme in nitrogen metabolism, asparaginase regulates nitrogen assimilation and partitioning in seeds, directly influencing seed development and size (62). The asparaginase-encoding gene *GhSID05* underwent positive selection during the second upland cotton domestication stage (divergence of the *latifolium* race) but was not targeted in the third stage (refinement of modern cultivars). Functional validation via virus-induced gene silencing (VIGS) further confirms GhSID05’s direct role in regulating seed traits. This aligns with our phenotypic data: seed index (SI), a key proxy for seed size, showed no significant divergence during the third domestication. Characterization of this overlooked seed yield locus provides insights for cotton genetic improvement, facilitating the development of dual-purpose varieties with enhanced lint and seed value.Our three-stage domestication framework reflects the combined effects of trait genetic architecture and stage-specific selection intensity: major-effect locus-regulated traits (e.g., seed index controlled by GhSID05) were rapidly fixed in single stages (D2), while polygenic traits (e.g., fiber quality, flowering time) required continuous selection across D2-D3 to accumulate minor-effect alleles, with genetic architecture being the core determinant of stage assignment.

Our research has decisively unveiled a fascinating evolutionary narrative regarding the elusive race *marie-galante*, shedding light on its origins through rigorous empirical investigation. Through comprehensive genetic analysis, we found compelling evidence of significant gene flow between *marie-galante* and *G. barbadense*. This finding has not only demystified the origins of race *marie-galante* but has also highlighted its intricate evolutionary trajectory. Furthermore, genomic studies have revealed a profound integration between *G. barbadense* and *G. hirsutum* race *marie-galante*, showcasing a mosaic-like dispersion of segments from *G. barbadense* throughout the genome. The MAR group identified in this study is an independent ecotype of *G. hirsutum* formed by introgression from *G. barbadense*. It has no genetic continuity with modern cultivated *G. hirsutum,* and its evolutionary process is independent of the three-stage domestication of *G. hirsutum*. This characteristic not only forms an effective complement to the previously reported *G. barbadense-G. hirsutum* introgression (8, 37, 63), but also does not contradict the core conclusion of the single origin of *G. hirsutum* in the Yucatán Peninsula; instead, it enriches the understanding of the postdomestication evolutionary and differentiation mechanisms of *G. hirsutum*.

These findings contribute valuable genetic resources to improve upland cotton through enhanced breeding strategies (13). Undeniably, a limitation of this study is that variant and haplotype analyses were anchored to the TM-1 reference genome, which may lead to mild reference bias. Lineage-specific structural and presence-absence variants unique to wild and semi-domesticated populations (e.g., YUC, PUN) could be underrepresented. However, our domestication inferences are mainly based on SNPs and population genetic statistics that are robust to such bias. Our main conclusions, therefore, remain valid. Future studies using cotton pan-genomes will help to resolve lineage-specific variants involved in early domestication.

## Materials and Methods

For detailed descriptions of sample selection, data generation, and analysis, see the *SI Appendix*. A total of 544 cotton accessions were sampled from the National Wild Cotton Germplasm Resources Nursery. The accessions were planted in four environments (2017 Xishuangbanna, 2017–2019 Sanya) with three biological replicates: Xishuangbanna (22°N) with an average daylength of 11.5 to 12.0 h during the growing season (May–October), and Sanya (18°N) with an average daylength of 11.0 to 11.8 h during the growing season (November–April). Phenotypic traits, including yield-related (boll weight, seed cotton weight, etc.), fiber quality (fiber length, strength, etc.), growth period (time to flower, boll opening, etc.), and morphological traits (stem trichomes, leaf shape), were measured following standardized protocols.

Genomic DNA was extracted using the CTAB method (64). Illumina Nova6000 sequencing generated ~15 Tb of data for 544 new samples. Clean reads were aligned to the Texas Marker-1 (TM-1) genome (CR1_v1: https://www.cottongen.org/node/13354433) (65) using BWA-MEM (v0.7.17-r1188 v0.7.17-r1188) (66), with variants called by GATK (v3.7.0) (67). Annotations were performed with SnpEff (4.3t) (68).

High-quality 4DTv SNPs (36,028) were used for maximum-likelihood phylogenetic tree construction (IQ-TREE) (v1.6.12) (69), PCA (PLINK, v1.9), and population structure analysis (ADMIXTURE, *K* = 2–10, v1.23) (70). Nucleotide diversity (π), *F*_ST_, and LD decay were calculated using VCFtools (v0.1.16, https://vcftools.github.io/index.html) (71) and PopLDdecay (v3.27) (72). Population dynamics were inferred using SMC++ (effective population size estimation, v1.15.4.dev18) (73) and fastsimcoal (nine demographic models, AIC-based selection, fsc27) (27). Introgressed genomic regions were retained in all analyses; Selection sweeps were detected via XP-CLR (top 1% windows) (74) across three domestication stages. *F*_ST_ analysis and DCMS method (15) were used for validation. ABBA-BABA test was applied to detect gene flow (75). Windows with <20 SNPs in 20 kb were excluded, with significance determined by *q*-value adjustment. GWAS was conducted on 15 traits for 432 accessions using 2,617,186 variants via EMMAX software (76). A global significance threshold of *P* < 1.0 × 10^−6^ was applied for most traits, with a refined threshold of *P* < 1.91 × 10^−7^ for SI, LM, and STR. Candidate genes were identified by LD block analysis and homologous annotation.

RNA-seq data from NCBI SRA were filtered (Dataset S15), and then aligned to TM-1 genome via HISAT2 (v2.2.1) (77), and gene expression quantified by featureCounts (v2.0.1) (78). GO enrichment analysis for candidate gene sets was performed using the R package ClusterProfiler (79) with Benjamini and Hochberg (BH) correction.

A *GhSID05* fragment (Dataset S16) was inserted into the CLCrVA vector, transformed into Agrobacterium GV3101, and infiltrated into CRI49 seedlings. Gene silencing efficiency was verified by RT-qPCR, with seed-related traits measured and analyzed via ImageJ and GraphPad Prism 9.5.0.

Statistical analyses were performed using software/tools including ADMIXTURE, fastsimcoal2, SMC++, PLINK, XP-CLR, and VCFtools. Methods included Bayesian models, maximum likelihood estimation, *t* tests, and FDR correction.

## Supplementary Material

Appendix 01 (PDF)

Dataset S01 (XLSX)

Dataset S02 (XLSX)

Dataset S03 (XLSX)

Dataset S04 (XLSX)

Dataset S05 (XLSX)

Dataset S06 (XLSX)

Dataset S07 (XLSX)

Dataset S08 (XLSX)

Dataset S09 (XLSX)

Dataset S10 (XLSX)

Dataset S11 (XLSX)

Dataset S12 (XLSX)

Dataset S13 (XLSX)

Dataset S14 (XLSX)

Dataset S15 (XLSX)

Dataset S16 (XLSX)

## Data Availability

Genomic and transcriptomic sequences data have been deposited in NCBI BioProject database under accession number (PRJNA1155012) (80).
