# Supplementary material for: Genomic reconstruction of upland cotton domestication uncovers staged selection, gene flow, and flowering-time adaptation
Source: Proc Natl Acad Sci U S A. 2026 Jun 22;123(26):e2601246123. doi: 10.1073/pnas.2601246123 (PMC13320693; doi:10.1073/pnas.2601246123)
Supplement: Supplementary file 1 — Appendix 01 (PDF) [file pnas.2601246123.sapp.pdf]

PNAS

Supporting Information for

Genomic reconstruction of upland cotton domestication uncovers staged selection, gene flow, and flowering-time adaptation

Yanchao Xu, Xiaoyan Cai, Zhongli Zhou, Damar Lopez-Arredondo, Yuqing Hou, Jie Zheng, Hongge Li, Gaofei Sun, Dingsha Jin, Panhong Dai, Yangyang Wei, Yuling Liu, Pengtao Li, Qiankun Liu, Heng Wang, Runrun Sun, Lijie Li, Xiaoping Pan, Kunbo Wang, Xiongming Du, Guoli Song, Baohong Zhang, Luis Herrera-Estrella, Shoupu He, Fang Liu, Renhai Peng.

Corresponding authors:
Baohong Zhang
Email: zhangb@ecu.edu
Luis Herrera-Estrella,
Email: luis.herrera-estrella@ttu.edu
Shoupu He,
Email: heshoupu@caas.cn
Fang Liu,
Email: liufang@caas.cn
Renhai Peng,
Email: 20160162@ayit.edu.cn

This PDF file includes:

Supporting text
Figures S1 to S21
SI References

Other supporting materials for this manuscript include the following:

None

Detailed Methods and Materials

Sampling and phenotyping of cotton accessions

A total of 544 cotton accessions were sampled for resequencing, including 65 accessions of *G. barbadense*, 39 accessions of *G. hirsutum* cultivars, and 440 accessions of *G. hirsutum* landraces. All samples were obtained from the National Wild Cotton Germplasm Resources Nursery (Cotton Research Institute, Chinese Academy of Agricultural Sciences) in Sanya, Hainan Province.

All 544 accessions were planted in four environments: during the winter of 2017 in Xishuangbanna, Yunnan Province, China (2017_BN), and over the winters of 2017-2019 in Sanya, Hainan Province, China (2017_BG, 2018_BG, and 2019_NB). Field management, including watering, weed and pest control, and fertilization, was performed in accordance with local cultivation standards. Each accession included three independent biological replicates from adjacent lots to ensure accurate data.

Standardized evaluation criteria were applied to all traits across all plots. Specifically, thirty uniformly developed bolls per line were hand-harvested from middle to upper branches to measure BW (g), SCW (g), SI (g), and LCW (g). SI was determined based on the weight of 100 cotton seeds. Lint percentage (LP, %) was calculated using SCW and LCW data (LP = LCW / SCW). Fiber quality traits, including FL (mm), fiber uniformity ratio (FU, %), FS (cN tex^{-1}), fiber elongation rate (FE, %), and FM, were assessed using HFT9000 (Premier Evolvics Pvt. Ltd., India) instruments with HVICC calibration in the Cotton Quality Supervision, Inspection and Testing Center, Ministry of Agriculture, Anyang, Henan Province, China.

In each biological replication, data on the dates for flowering, boll formation, and flower opening were recorded daily. TOF, TOB, and TOOB were calculated as the duration from sowing to the first appearance of flowers, bolls, and opened bolls in 50% of the plants in each line, respectively. The node of the first fruiting branch (NFFB) for 10 individual plants per line was recorded, and the mean of these records, after removing outliers, was used as the NFFB trait data. In each replication, daily records were kept of flowering, boll, and opened-boll dates.

Stem trichomes (STR) were quantified by counting the number of long trichomes on one side of the stem within the frame using a dissecting microscope (OLYMPUS DP27, 1× objective lens, 0.68 magnification). Leaf morphology was visually assessed across all accessions using predefined categories: broad leaf (scored as 1), palmate leaf (scored as 2), and okra leaf (scored as 3), as previously reported(1, 2). All phenotype data were processed as the best linear unbiased prediction (BLUP) values across three replications and four environments using the R package ‘lme4’ (https://github.com/lme4/lme4)(3).

Sequencing/Resequencing, alignment and SNP calling

Genomic DNA from the 544 cotton accessions was extracted using the CTAB method(4). Paired-end Illumina libraries (150-bp reads, ~350-bp insert size) were constructed and sequenced on the Illumina Nova6000 platform, generating approximately 15 terabases (Tb) of data with a 10× depth per accession. The data was filtered to remove adaptor sequences and low-quality reads (>10% unknown bases or average quality <20). Additionally, 2,429 samples from previous resequencing were downloaded, but 63 were excluded due to duplication or insufficient depth(5, 6). In total, 97.53 TB of raw sequencing data, with an average coverage of 16x, was used for variant calling. Clean reads were aligned to the Texas Marker-1 (TM-1) genome (CR1_v1: https://www.cottongen.org/node/13354433)(7) using the Burrows–Wheeler Aligner MEM (BWA-MEM, v0.7.17-r1188, default settings)(8), with alignments sorted via Samtools (v1.51.1)(9) and duplicates marked using sambamba (v0.8.2)(10). The HaplotypeCaller module in GATK (v3.7.0)(11) was used to generate gVCF files for each accession. Subsequently, all individual gVCF were combined using CombineGVCFs MODULE IN GATK, and joint genotyping was performed on the combined dataset with the GenotypeGVCFs module to produce the final integrated VCF file. The quality of vcf file was hard filtered using VariantFiltration (for SNP: QD < 2.0 || FS > 60.0 || MQ< 40.0 || SOR > 3.0 || MQRankSum < -12.5 || ReadPosRankSum < -8.0; for Indels: QD < 2.0 || FS > 200.0 || SOR > 10.0 || InbreedingCoeff < -0.8 || ReadPosRankSum < -20.0). The missing rate and heterozygosity rate for each sample were calculated using PLINK (v1.9(12). Final variant maps were filtered for minor allele frequency (>5%) and missing data (<20%), resulting in a high-quality SNP and InDel dataset for 2,910 accessions. Annotations were completed using SnpEff (4.3t)(13).

GWAS analysis

Genotype data from 432 cotton accessions were analyzed using GWAS with 2,617,186 variations filtered from 2,910 accessions, applying a MAF > 0.05 and a MISSING RATE < 0.2. GWAS was performed on 15 traits using EMMAX software(14), incorporating a kinship (K) matrix to correct for population structure. Significance was determined with $p < 1.0 \times 10^{-6}$. LDblockShow (v.1.32, https://github.com/BGI-shenzhen/LDBlockShow)(15) assessed local linkage disequilibrium in candidate regions. Candidate genes were identified by sorting SNPs and Indels within LD blocks by *p*-values ($<10^{-5}$), and gene annotations were guided by evidence from Arabidopsis thaliana or other plants. Haplotype analysis linked haplotypes to phenotypes to confirm genetic effects. Additionally, candidate gene protein sequences were compared with Arabidopsis thaliana proteins using BLASTP (BLAST+, v2.5.0)(16), and 2 kb upstream sequences of protein-coding genes were analyzed for regulatory elements using SeqKit (v0.15.0)(17) and the Plant CARE database (http://bioinformatics.psb.ugent.be/webtools/plantcare/html/)(18).

Phylogenetic, population structure and population diversity analyses

Bi-allelic SNPs were filtered using bcfools (v1.11)(9) with criteria FORMAT/DP < 5, FORMAT/GQ < 30, F_MISSING > 0.1, and MAF < 0.05 for phylogenetic and population structure analyses. SNPs at fourfold-degenerate sites (36,028 SNPs) were used to construct a maximum-likelihood phylogenetic tree with IQ-TREE (v1.6.12)(19), employing the MFP parameter for substitution model optimization and 1,000 bootstrap replicates. To generate 4DTv (fourfold degenerate synonymous sites) SNPs, we initiated the process by extracting coding sequences (CDS) from the *G. hirsutum* reference genome TM-1 (CR1_v1: https://www.cottongen.org/node/13354433)(7). Subsequently, a custom Perl script was employed to precisely identify the 4DTv sites. We finally employed VCFtools (v0.1.16, https://vcftools.github.io/index.html)(20) with the "--positions" flag to extract SNPs located within these regions. The tree was visualized with the R packages ape and ggtree. Principal component analysis (PCA) was performed using PLINK (v1.9), and population structure was analyzed with ADMIXTURE (v1.23)(21), running analyses 20 times for K values ranging from 2 to 10. Nucleotide diversity (π) and fixation statistic (F_{ST}) for 2,910 accessions were calculated using VCFtools(20), with average FST computed in 50 kb windows. Linkage disequilibrium (LD) decay for SNP pairs within 500 kb was assessed using PopLDdecay (v3.27)(22).

Demographic inference

Recent population dynamics were inferred using SMC++ (v1.15.4.dev18)(23), based on multi-sample resequencing data. Pseudo-diploid genotypes were generated from whole-genome sequencing (WGS) data and compiled into a single VCF file. Effective population sizes (*N*e) were estimated for each group using WGS data from randomly selected samples (n=20 per dataset) and SMC++, with a generation interval of 1 and a mutation rate (μ) of 3.61×10^{-9}.

Additionally, fastsimcoal (fsc27), a coalescent simulation-based method that utilizes a folded site frequency spectrum (2D-SFS), was employed to reconstruct the population history(24). The site frequency spectrum (SFS) was computed using the Python script easySFS.py (available at https://github.com/isaacovercast/easySFS). Nine distinct models were formulated to estimate parameters, including population divergence times, effective population sizes, and migration rates. Each model underwent 100 estimation runs with fastsimcoal, each run comprising 100,000 coalescent simulations (-n 100,000) and 50 rounds of conditional expectation maximization cycles (-L 50). The mutation rate (μ) was set at 3.61×10^{-9}. Model selection was conducted using the Akaike information criterion (AIC), with the optimal model chosen based on the lowest AIC value(25). The formula for AIC is: AIC = 2k-2ln (MaxEstLhood), where k is the number of parameters for each model, and MaxEstLhood is the maximum likelihood function value for each model.

Identification of selection sweeps

We used the cross-population composite likelihood ratio test (XP-CLR, v1.1)(26) to detect selection signals during three domestication events. We identified selective sweeps in four groups (Cul1 *vs.* Lat, Cul2 *vs.* Lat, Lat *vs.* Pun, and Pun *vs.* Yuc), with the first two groups representing the third domestication of upland cotton, and the latter two representing the second and first domestications of upland cotton, respectively. We used XP-CLR to compute scores for each chromosome with the parameters "--ld 0.95 --maxsnp 300 --size 50000 --step 5000". We selected windows with XP-CLR scores in the top 1% as candidate selective sweep regions. Additionally, the F_{ST} values for four pairwise comparisons with a 50kb window size and a 5kb step were used to detect population differentiation. We standardized the F_{ST} to obtain Z-F_{ST} scores using the following formula: Z-F_{ST} = ($F_{ST} \times F_{ST}$)/std-F_{ST}, where F_{ST} is the value for each window, F_{ST} and std-F_{ST} are the mean and standard error of all F_{ST} values for all windows, respectively. Then, we used a student's t-test to evaluate the null hypothesis that the F_{ST} value for each window equals the average F_{ST} value for all windows. The resulting *p*-values were corrected using the false discovery rate (FDR). We considered windows with F_{ST} >2 and corrected *p*-value <0.001 as significantly high-differentiation outlier windows. Then, we merged adjacent outlier windows (with an interval less than 20kb) into larger windows using bedtools(27). Finally, we integrated F_{ST} and XP-CLR statistics using the DCMS method(28). First, F_{ST} and XP-CLR values were converted to one-tailed *p*-values (right-tailed for both) using stat_to_pvalue from the R package MINOTAUR. Next, the covariance matrix of these *p*-values was estimated via CovNAMcd (alpha = 0.75 and nsamp = 10,000) in rrcovNA, accounting for statistical correlations. This matrix was then input into MINOTAUR's DCMS function to compute composite signals. DCMS values were normalized into a pseudo-normal distribution using robust linear regression (rlm in MASS), and their *p*-values were converted to FDR-adjusted *q*-values via qvalue(29). Regions with $q < 0.05$ were deemed significant selective sweeps, integrating evidence of F_{ST} and XP-CLR for enhanced reliability(30).

Identification of introgression

The ABBA-BABA test with a 4-taxon topology was employed to assess gene flow between populations(31). Specifically, the D-statistic was utilized to examine the imbalance between ABBA and BABA site patterns, indicating the level of introgression averaged across genomic regions of a specified length. In this analysis, an outgroup (O) was used alongside three ingroup taxa: Pun (P1), allopatric Mar (P2), and Gb (P3), structured as (((P1, P2), P3), O). In the absence of gene flow, ABBA and BABA patterns are expected to occur equally due to incomplete lineage sorting. However, if there has been gene flow between P2 and P3, an excess of ABBA patterns relative to BABA is anticipated. Detection of introgression at the genome-wide level using the Python script 'ABBABABAwindows.py' (https://github.com/simonhmartin/genomics_general/ABBABABAwindows.py). Windows containing fewer than 20 SNPs within 20 kb were excluded from analysis. The significance of the

D-statistic for each window was determined based on the normal distribution with adjustments for multiple testing effects using the R package 'qvalue' (*q*-value).

RNA-seq and expression differentiation analyses

RNA-seq data were downloaded from NCBI SRA database **(SI Appendix, Table S15)**, included tissues representing the fiber cell initiation stage (ovules at –3, 0 and 3DPA), fiber cell elongation stage (ovules and fiber at 5, 10 and 15DPA) and fiber cell secondary wall synthesis stage (ovules and fiber at 20 and 25DPA). The raw data were first filtered using fastq. Then, clean data were mapped to reference genome TM-1 (CR1_v1: https://www.cottongen.org/node/13354433)(7) using hisat2 (v2.2.1)(32). Gene expression was calculated using featureCounts (v2.0.1) software(33).

Gene ontology analysis

All gene annotation information was obtained from the reference genome TM-1 (CR1_v1: https://www.cottongen.org/node/13354433)(7). The candidate gene sets were analyzed using R package ClusterProfiler(34) for gene ontology (GO) enrichment analysis. Benjamini and Hochberg (BH) correction was used to adjust significance levels for multiple comparisons.

Virus-Induced Gene Silencing (VIGS)

To clarify *GhSID05*'s role in upland cotton seed index, *G. hirsutum* cv. CRI49 were used. A 302-bp *GhSID05* fragment (**SI Appendix, Table S16**) was inserted into CLCrVA vector to generate *CLCrVA:GhSID05*, with empty *CLCrVA:00* as control. Both constructs were transformed into *Agrobacterium tumefaciens* GV3101. Cotyledon-stage seedlings of CRI49 were infiltrated with *Agrobacterium* following a standard cotton VIGS protocol(35), then cultured at 25±1℃ (16 h light/8 h dark, 65–70% humidity). At the three-true-leaf stage, leaf RNA was extracted (TIANGEN DP432 Kit), reverse-transcribed to cDNA (Takara RR047A Kit), and qRT-PCR was performed to detect *GhSID05* expression. Silencing efficiency was quantified via $2^{-\Delta\Delta Ct}$ method (internal control: *Ghactin*). Mature seeds from VIGS and wild-type (WT) plants were de-fuzzed for phenotyping. Seed images (with ruler scale) were analyzed via ImageJ to measure length, width, and area; weight was determined with a 0.1 mg balance. Data were analyzed/visualized using GraphPad Prism 9.5.0, with significance tested by Student's *t*-test.

Data Statistics

The statistical analysis methods involved in this study include various computer programs and tools, such as ADMIXTURE, fastsimcoal2, SMC++, PLINK, XP-CLR, VCFtools, and R package ClusterProfiler. These tools and programs use various statistical models and algorithms, including Bayesian models, maximum likelihood estimation, integrated likelihood methods, t-tests, False

Discovery Rate (FDR) correction, D-statistic, π ratio, F_{ST}, and GO enrichment analysis. We strictly followed the data preprocessing, parameter setting, and result interpretation instructions for each tool and program to ensure the accuracy and reliability of the results. For complex analysis pipelines, we performed repeated validation and cross-checks to ensure reliable and meaningful results. Additionally, we provided detailed descriptions and explanations of each analysis method.

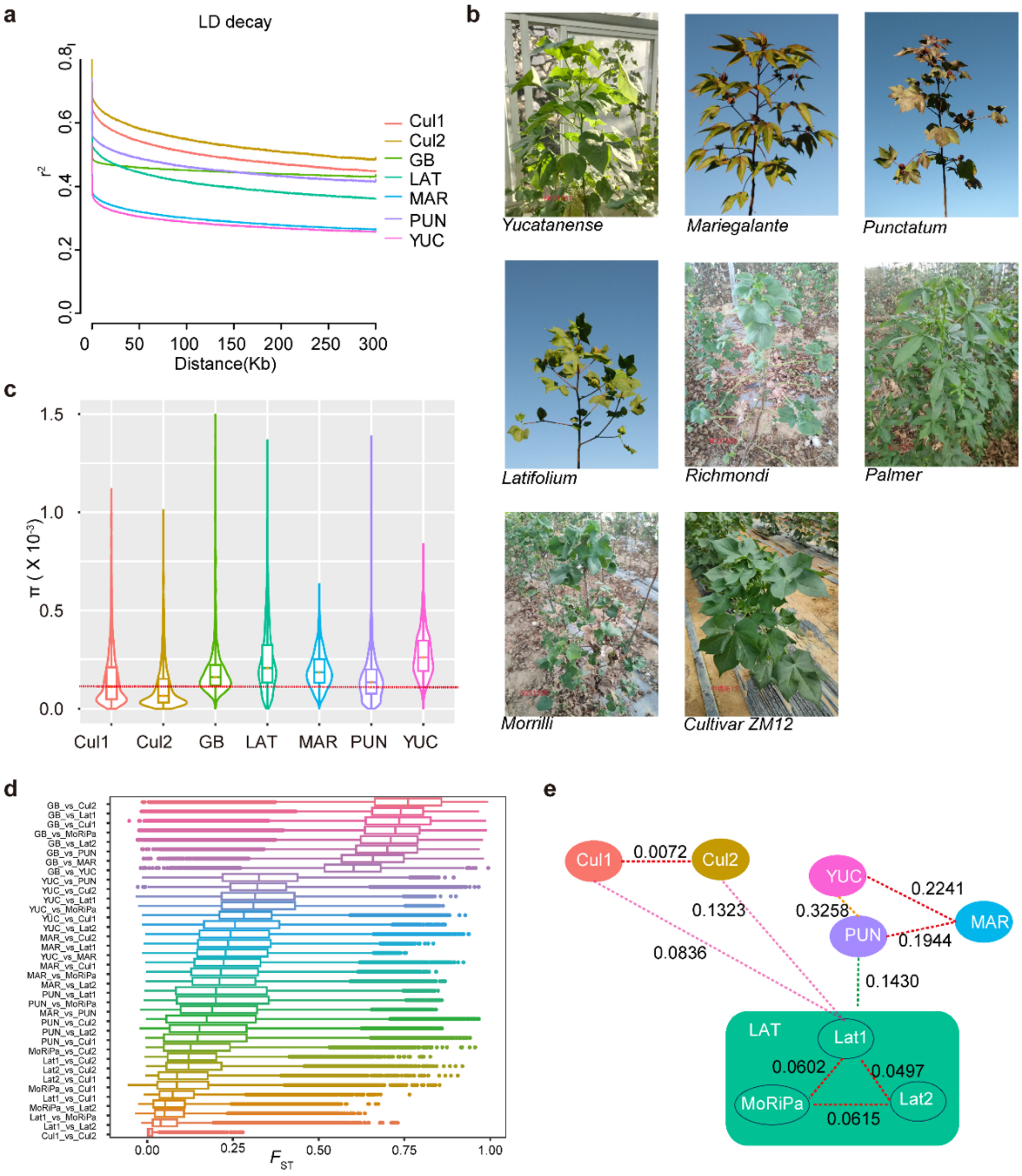

Fig. S1. The phenotype and genetic divergence of *G. hirsutum* races. a, Linkage disequilibrium (LD) decay pattern across different *Gossypium* groups. **b,** The morphological characteristics of seven *G. hirsutum* race at three months of age. **c,** Boxplots depicting nucleotide diversity (π) among different *G. hirsutum* groups. For each box plot, the lower and upper bounds indicate the first and third quartiles, respectively, with the center line representing the median. **d,** The comparison of pairwise F_{ST} values across different populations of *G. hirsutum*. **e,** the average pairwise F_{ST} values between different populations are represented along the line.

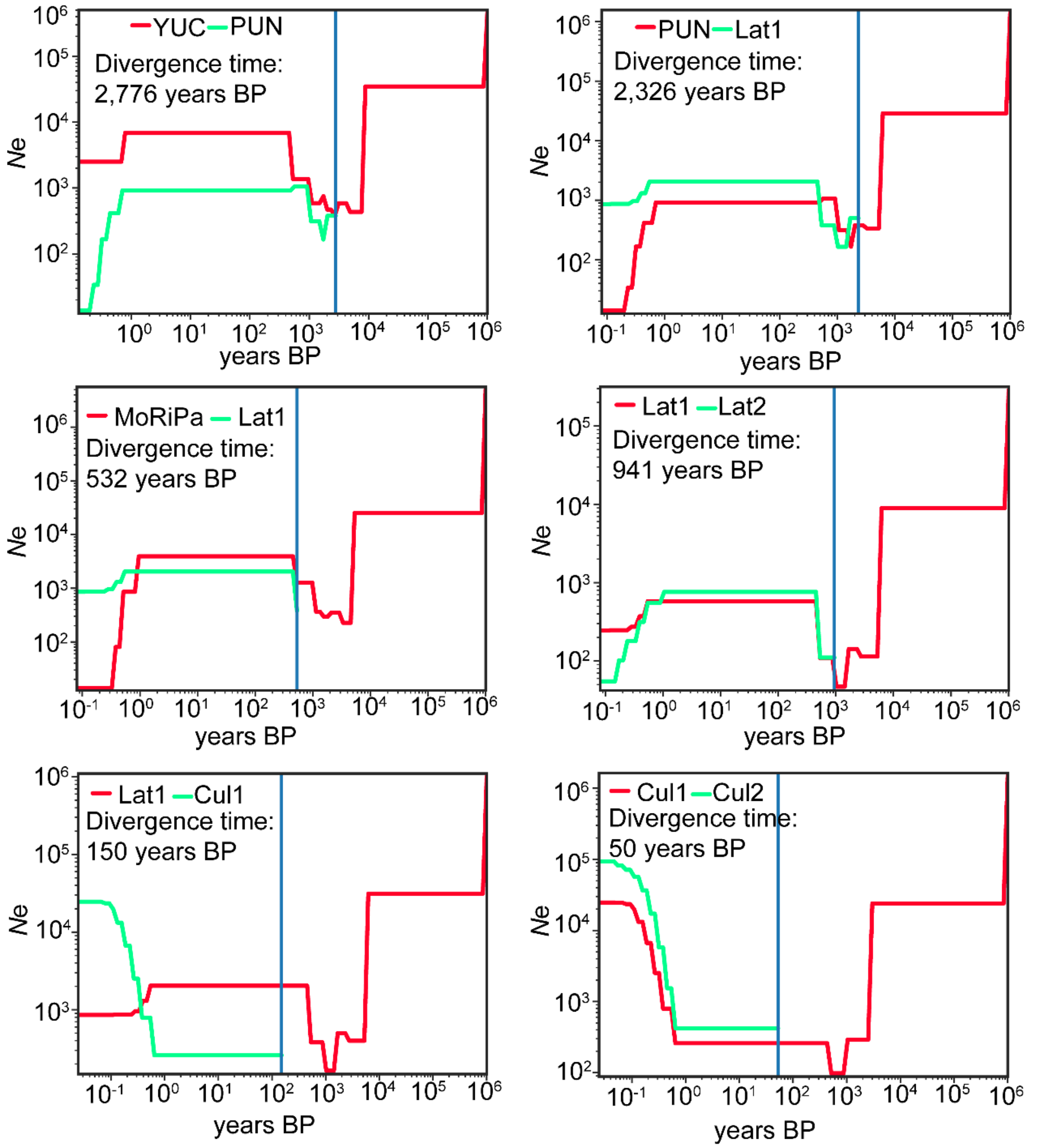

Fig. S2. The population divergence times estimated using smc++ software. Generation time: 1; Mutation rate: 3.61×10^{-9}.

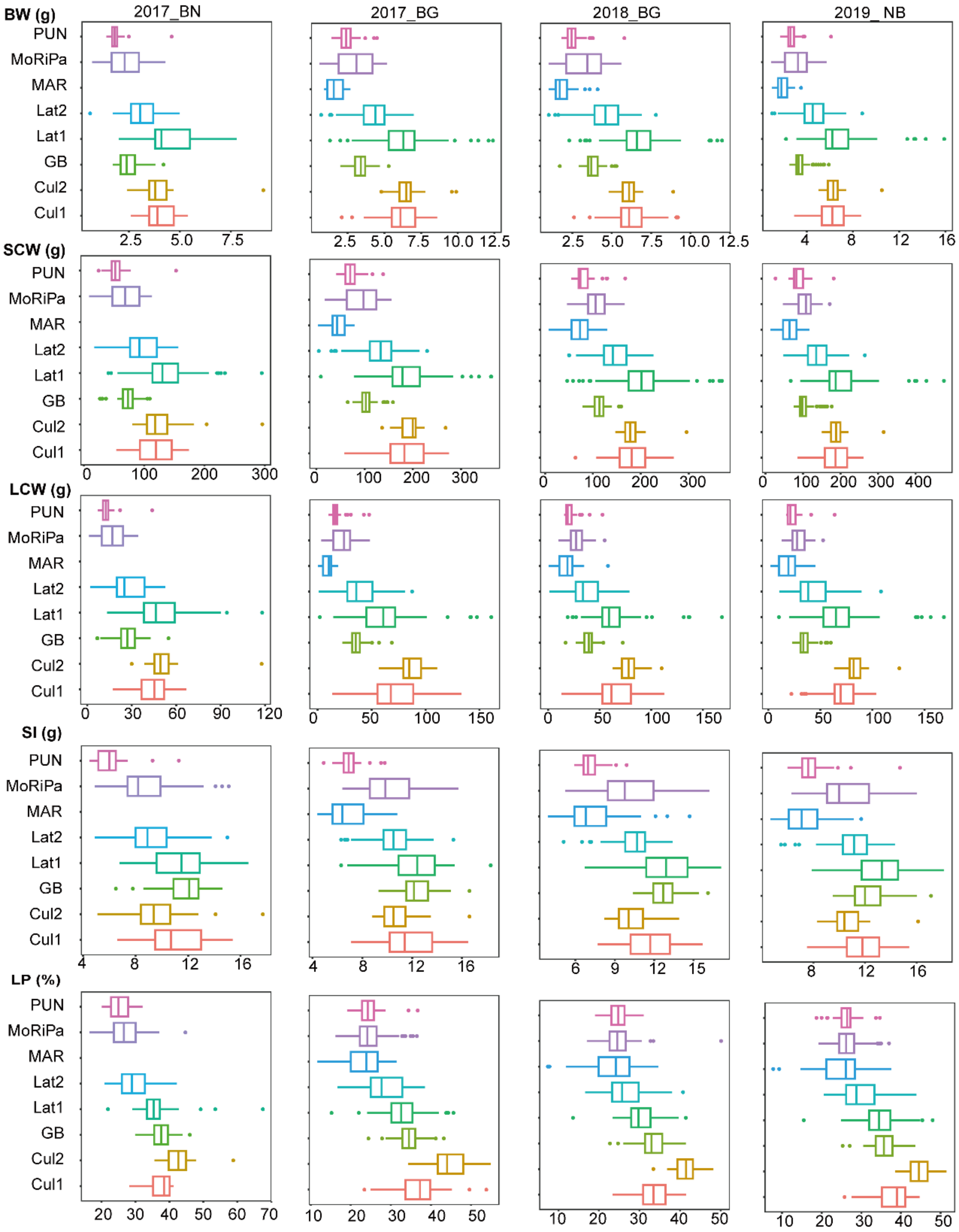

Fig. S3. The distribution characteristics of yield traits in different populations of *G. hirsutum* across four growth environments. The MAR group did not flower during the growing season in Xishuangbanna, hence yield phenotype data were unavailable. Similarly, the YUC group did not flower in any region, resulting in missing phenotype data.

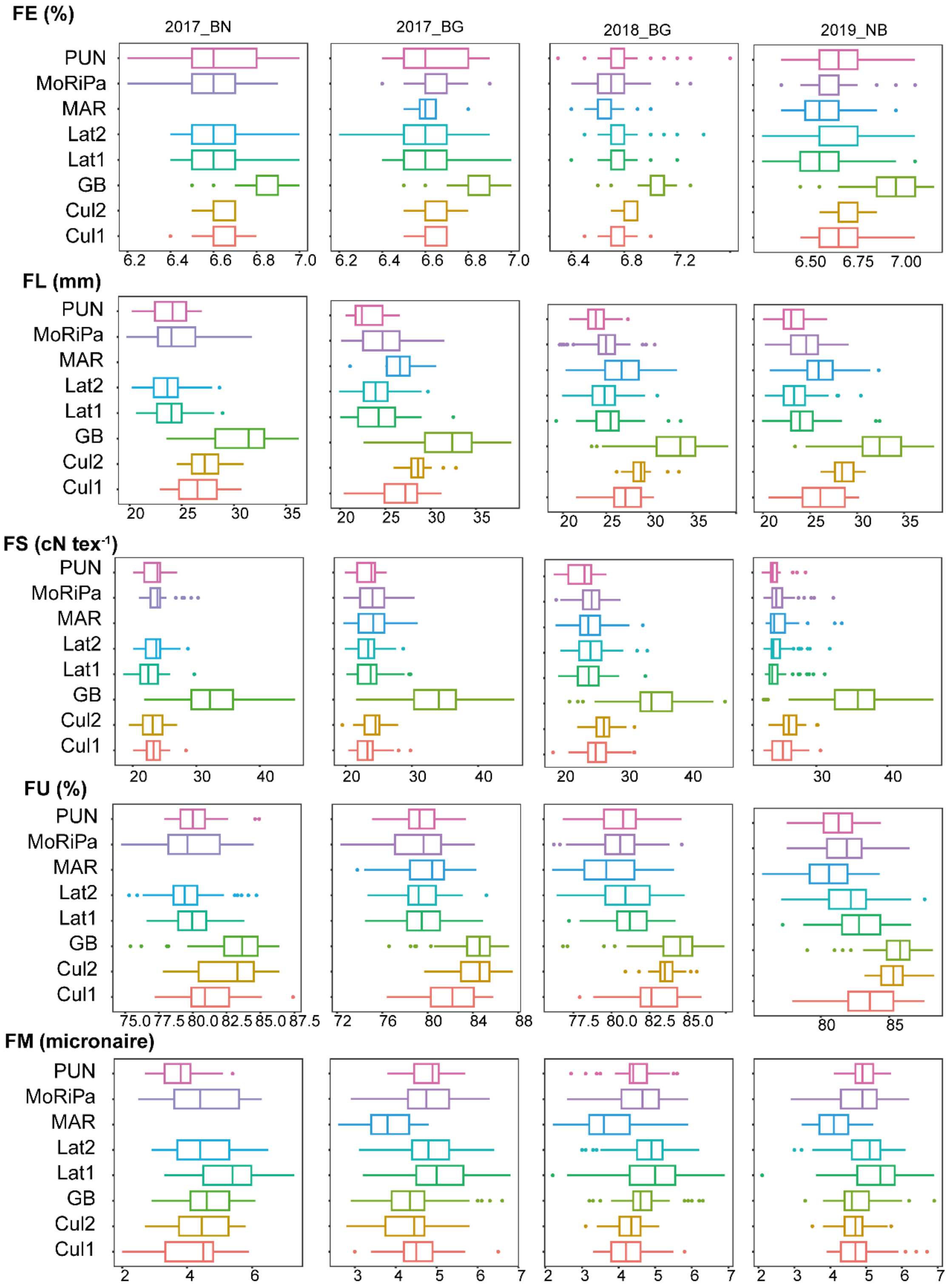

Fig. S4. The distribution characteristics of fiber traits in different populations of *G. hirsutum* across four growth environments. The MAR group did not flower during the growing season in Xishuangbanna, hence yield phenotype data were unavailable. Similarly, the YUC group did not flower in any region, resulting in missing phenotype data.

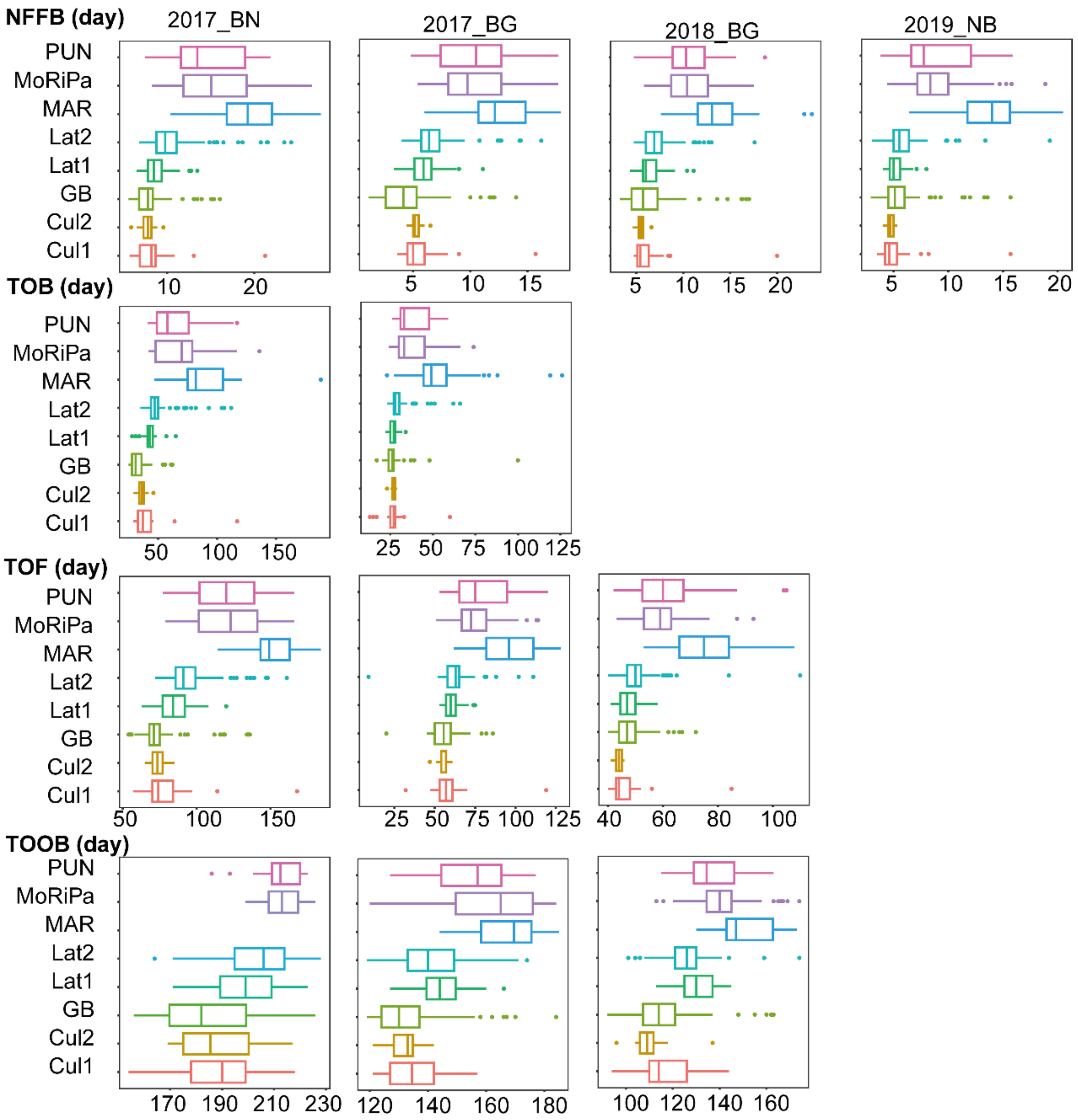

Fig. S5. The distribution characteristics of growth stages in different populations of *G. hirsutum* across four growth environments. The MAR group did not flower during the growing season in Xishuangbanna, hence yield phenotype data were unavailable. Similarly, the YUC group did not flower in any region, resulting in missing phenotype data.

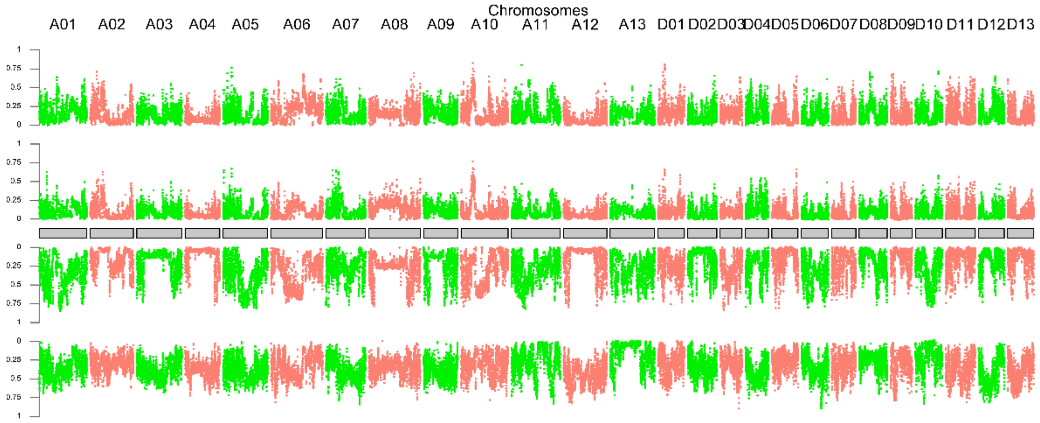

Fig. S6. Genome-wide selective sweep analyses using F_{ST} across five groups (YUC, PUN, LAT, and Cul1/Cul2).

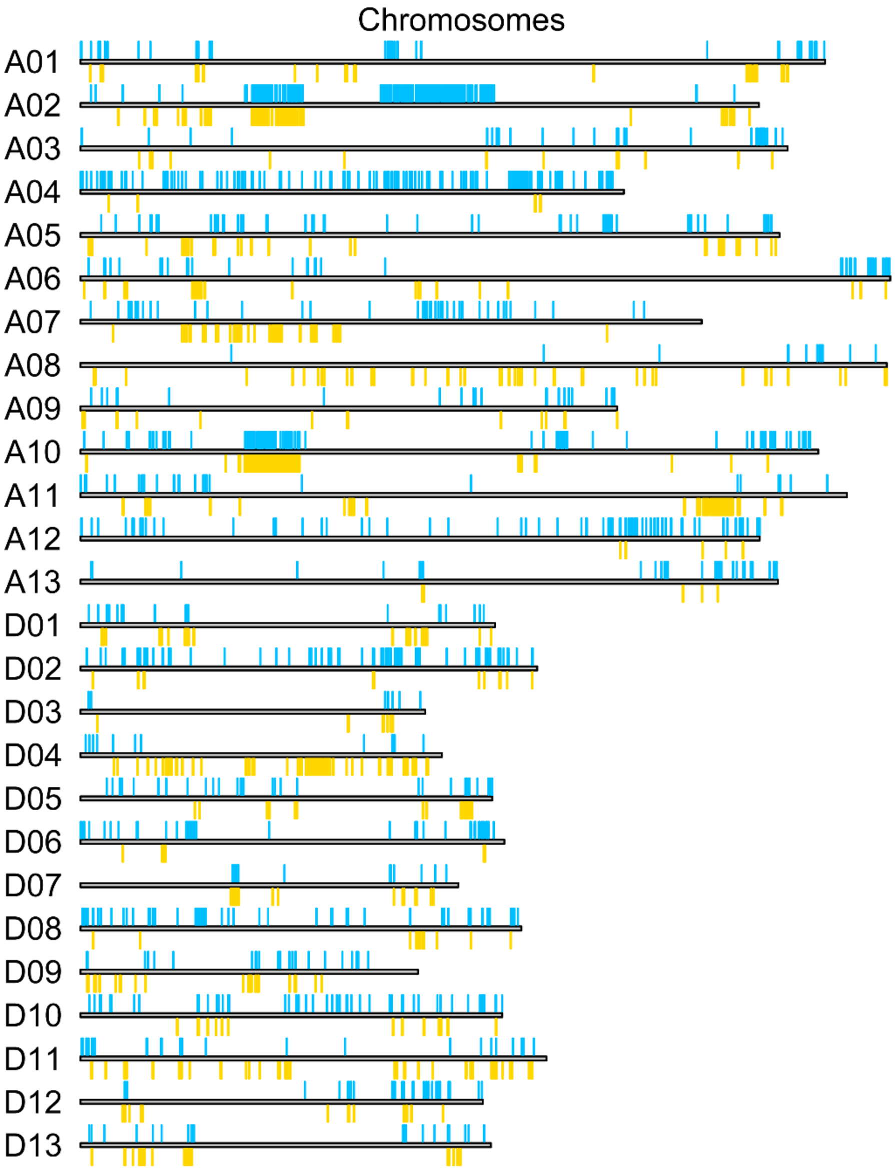

Fig. S7. The selective sweep regions identified used xpclr (blue) and F_{ST} (gold) between Cul1 vs. Lat1.

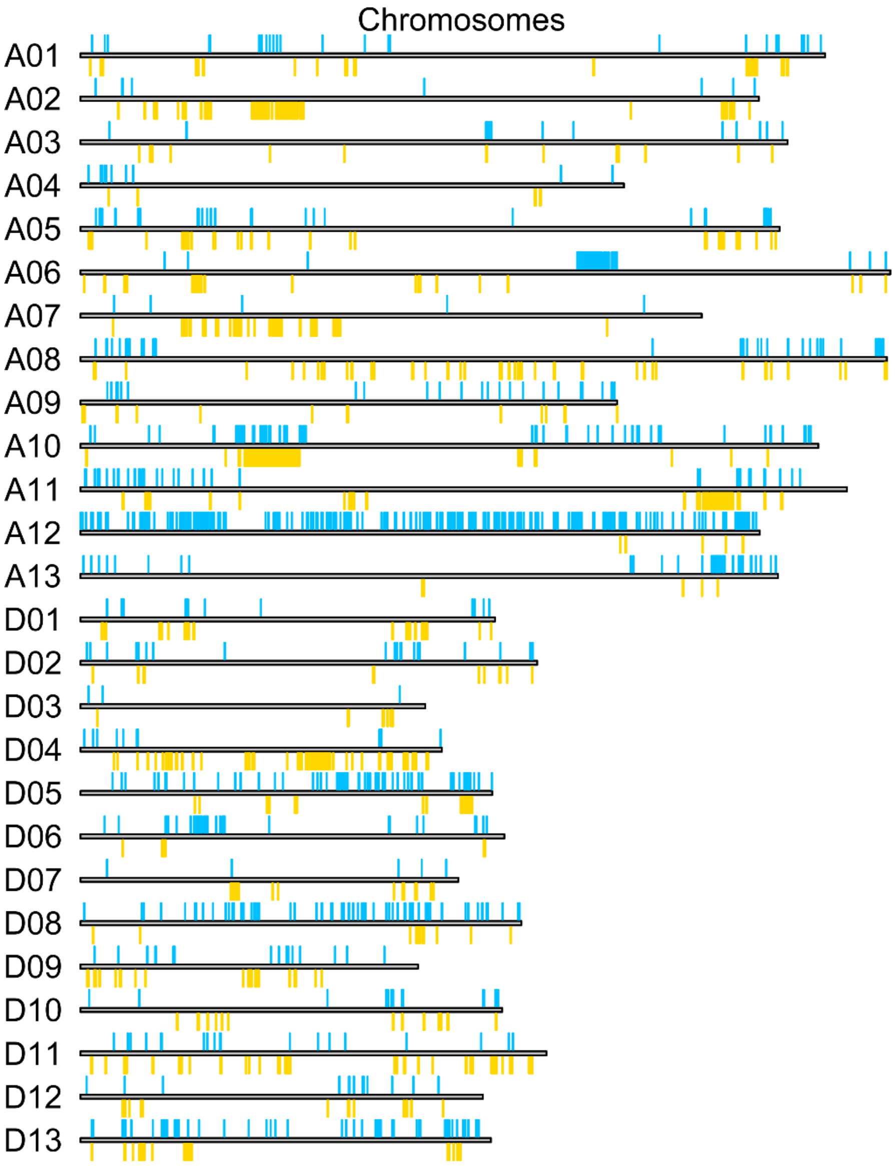

Fig. S8. The selective sweep regions identified used xpclr (blue) and F_{ST} (gold) between Cul2 vs. Lat1.

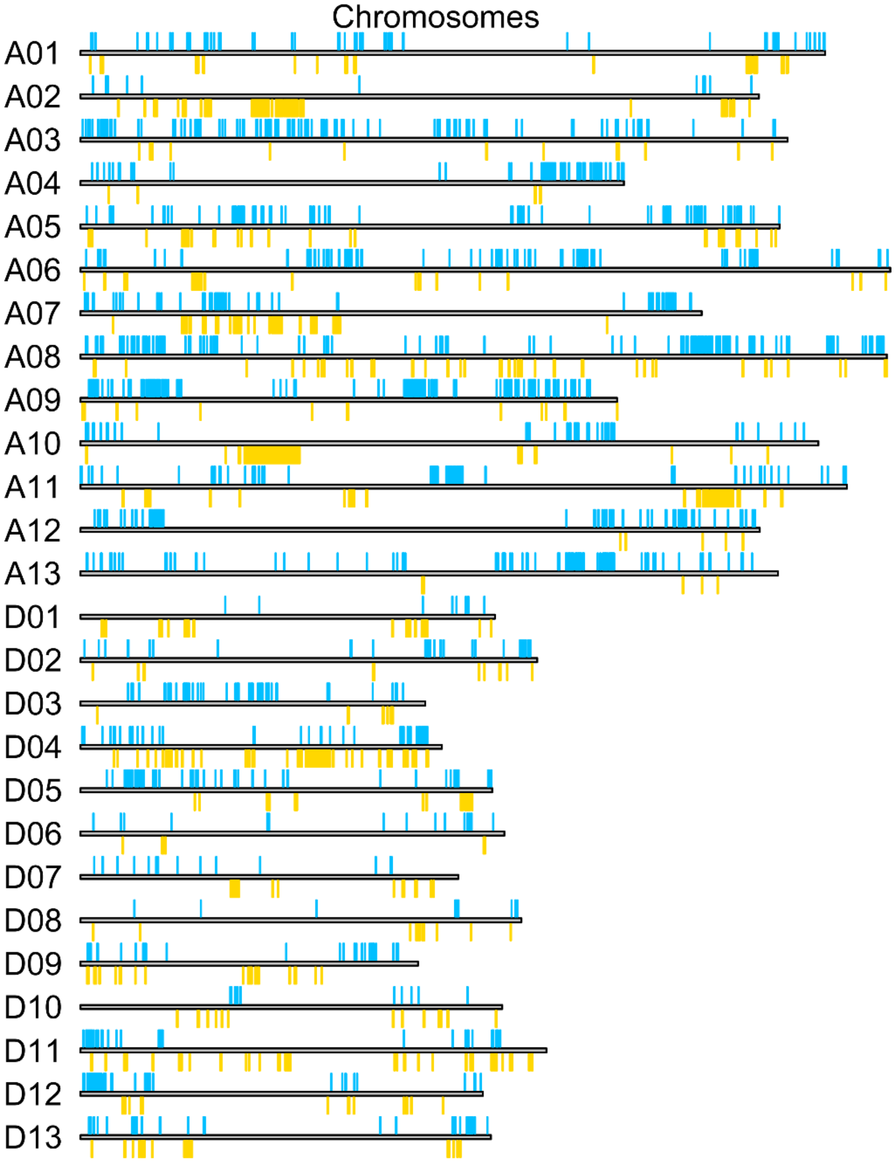

Fig. S9. The selective sweep regions identified used xpclr (blue) and F_{ST} (gold) between Lat1 vs. PUN.

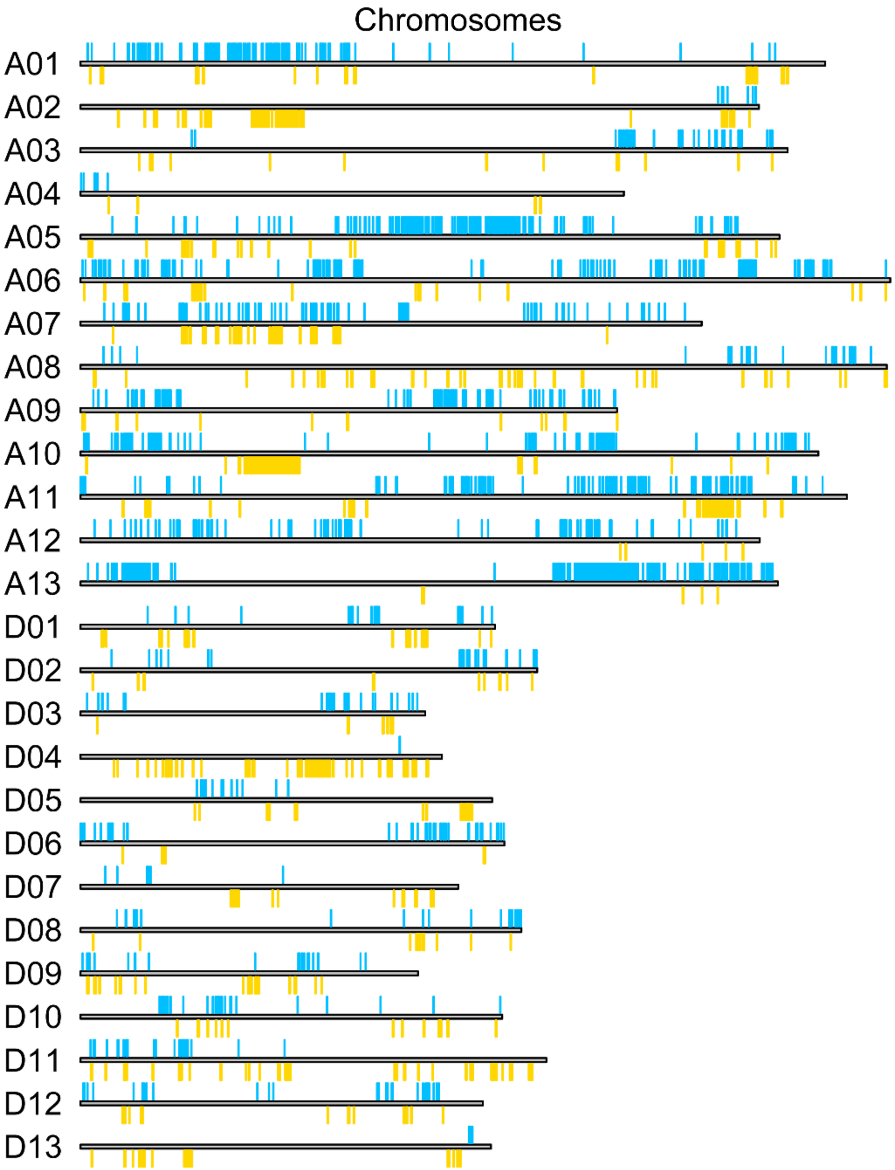

Fig. S10. The selective sweep regions identified used xpclr (blue) and F_{ST} (gold) between PUN vs. YUC.

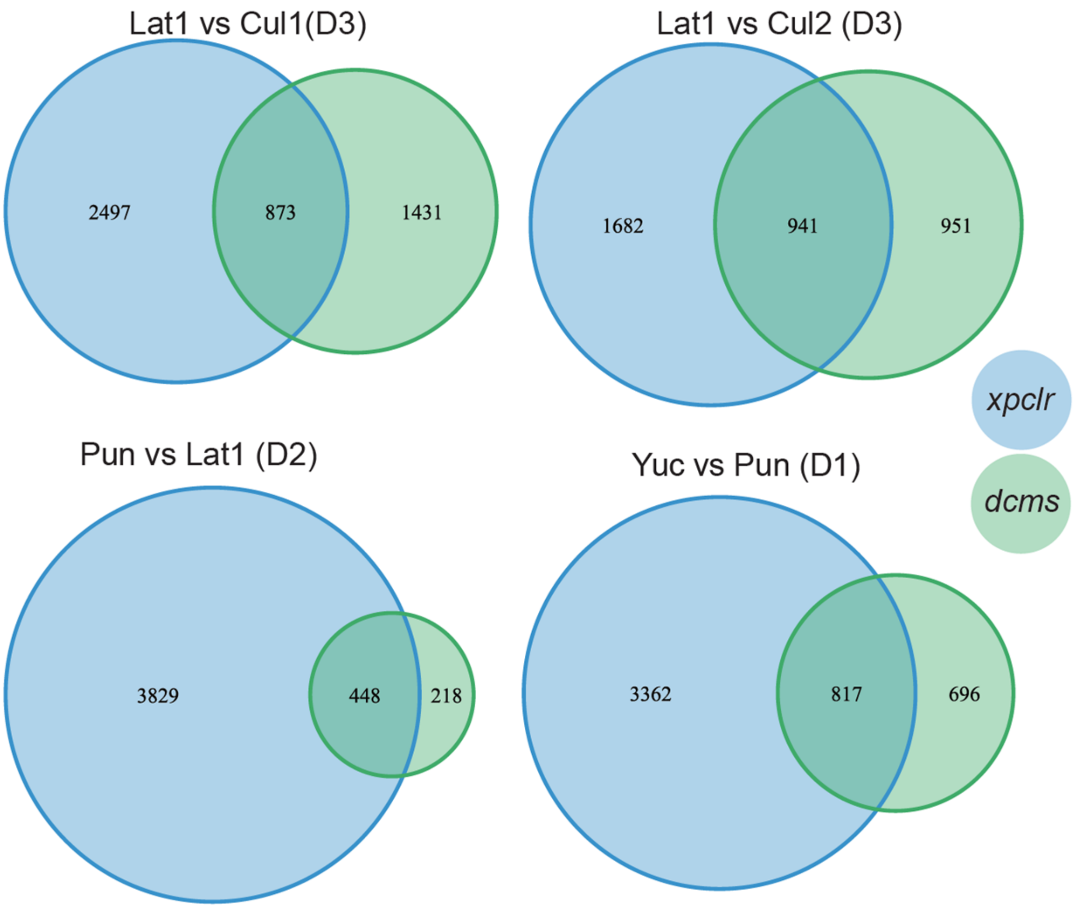

Fig. S11. The overlap of selective sweep regions between dcms and xpclr.

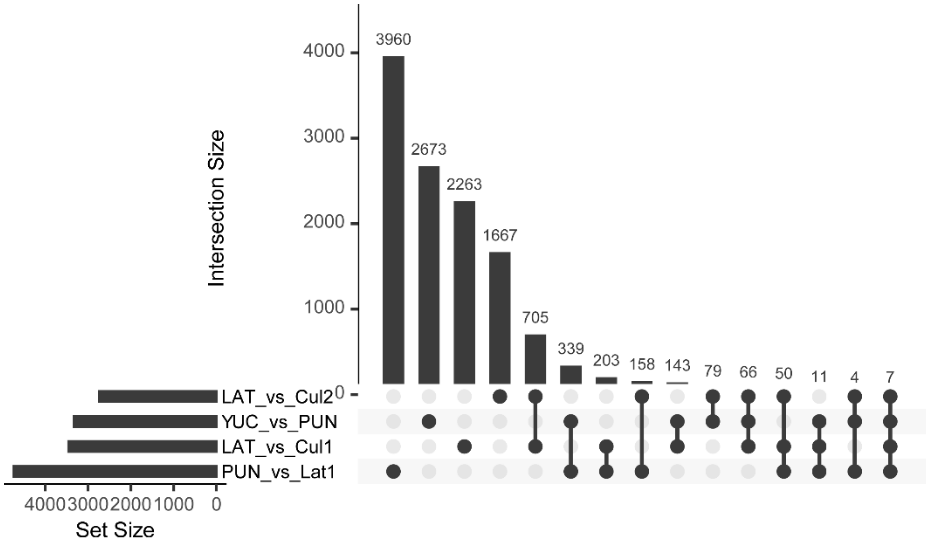

Fig. S12. The UpSet plot illustrates shared and uniquely selected sweep signals across three domestication events.

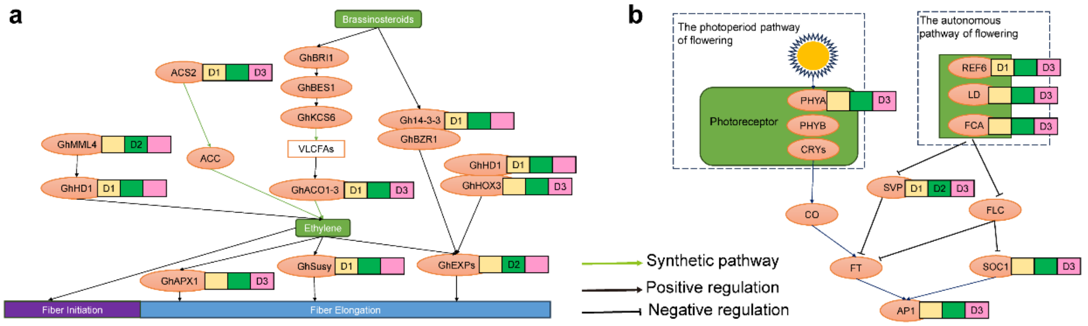

Fig. S13. Gene regulatory network for cotton fiber and flowering. a, Regulatory networks for cotton fiber initiation and elongation including Brassinosteroid- and ethylene- mediated regulatory networks. **b**, Regulatory networks for flowering including the photoperiod pathway and autonomous pathway. For **a** and **b**, mark the genes overlapped with signatures of selective sweeps in boxes behind the gene names.

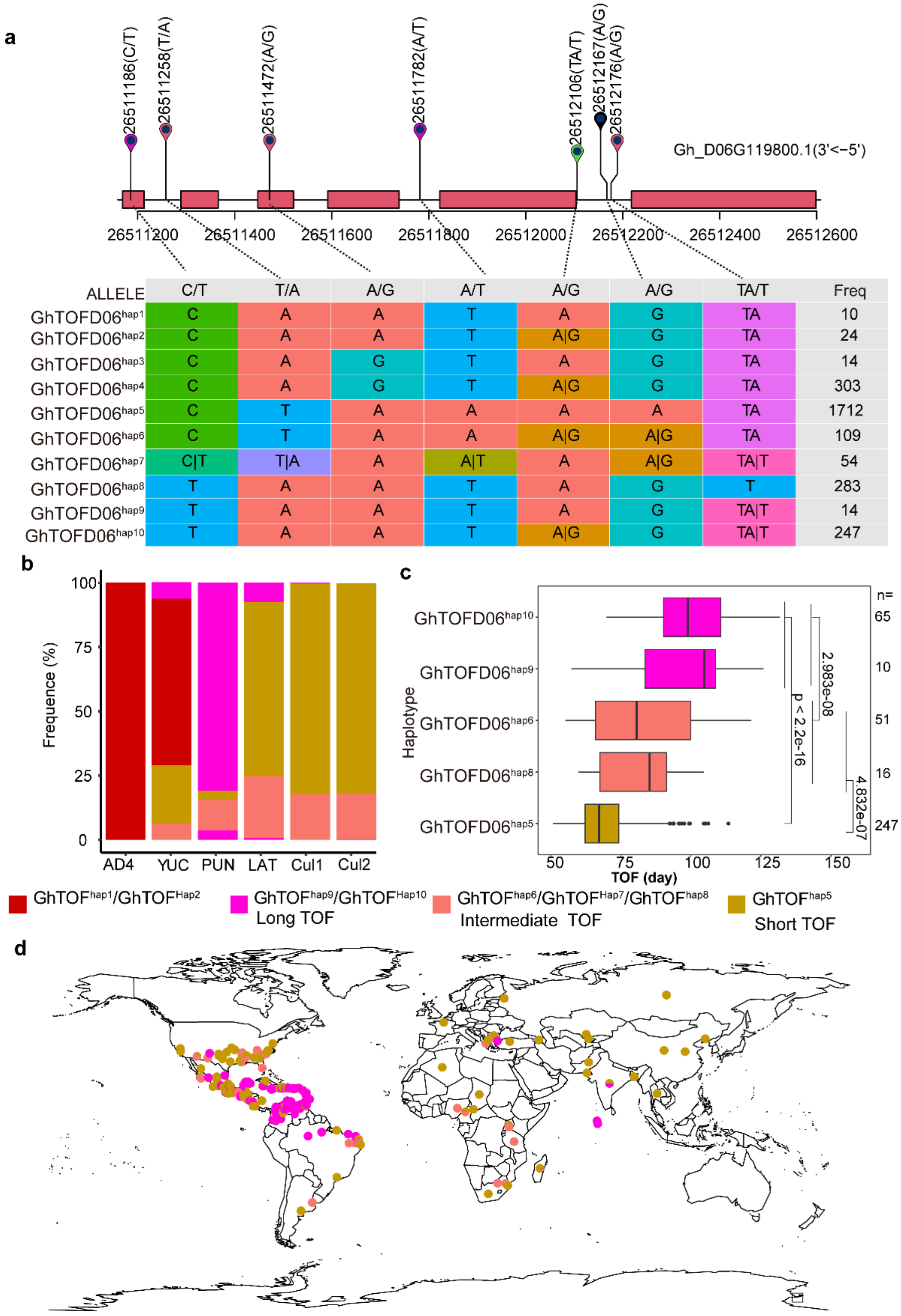

Fig. S14. Genotype and evolution the candidate gene *GhTOFD06*. a, Gene structure and the variation site of *GhTOFD06* (top), the major different haplotypes of *GhTOFD06* (bottom). **b,** The haplotype frequency of *GhTOFD06* in different cotton populations. **c,** The quantification of TOF of five haplotypes in *G. hirsutum*. Statistical hypothesis test: two-tailed t-test. **d,** Geographic distributions of ten haplotypes.

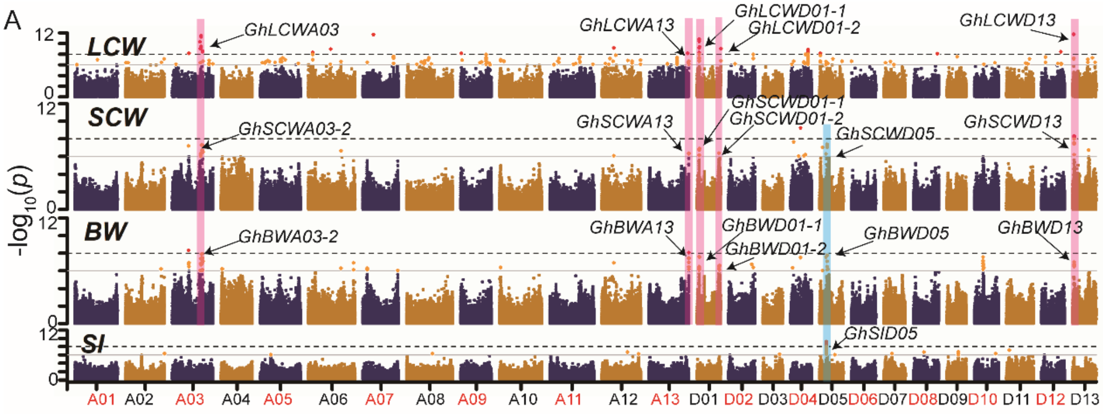

Fig. S15. Manhattan showed the yield traits based on GWAS. The horizontal dashed line represents the significance threshold (P< 1 × 10^{-6} and P<1 × 10^{-8}, Bonferroni correction).

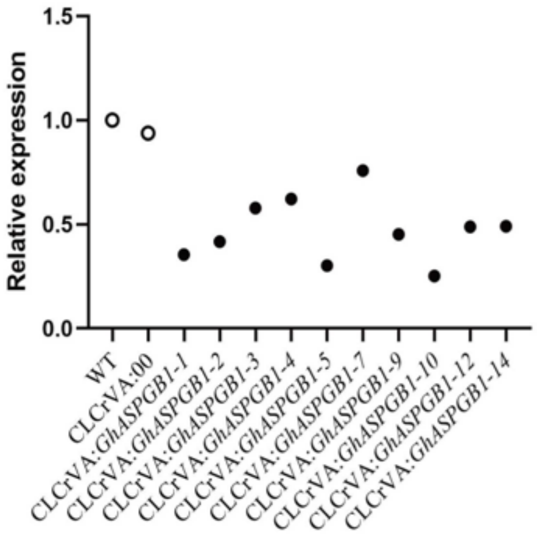

Fig. S16. Virus-induced gene silencing (VIGS) of *GhSID05* (*GhASPGB*) in CRI49. The Relative expression of WT, CLCrVA:00 and CLCrVA:GhSID05 plants.

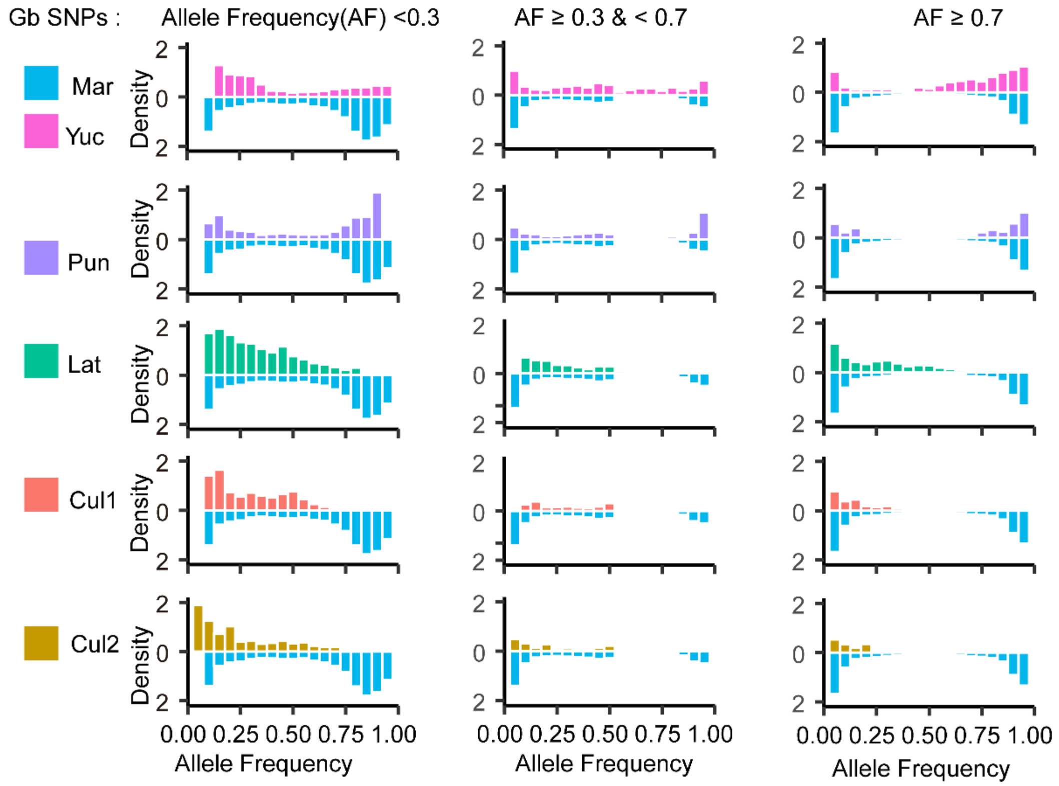

Fig. S17. The frequency distribution of alternative variant sites in different groups, where alternative allele frequencies < 0.3, ≥ 0.3 &<0.7, ≥ 0.7 in *G. barbadense*, respectively.

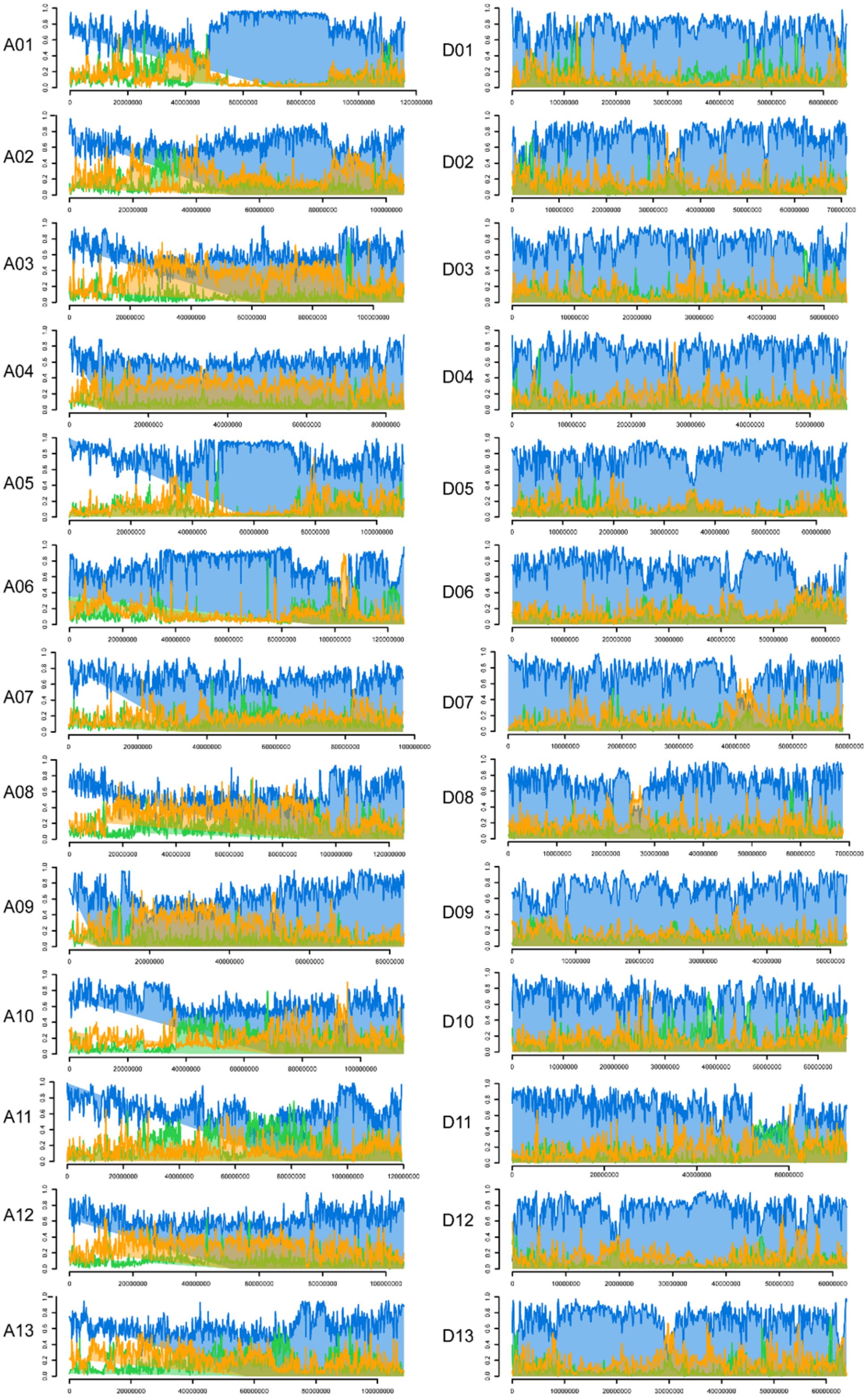

Fig. S18. Distribution of average weights for all three topologies described in **Figure 2c** across 26 chromosomes using sliding windows.

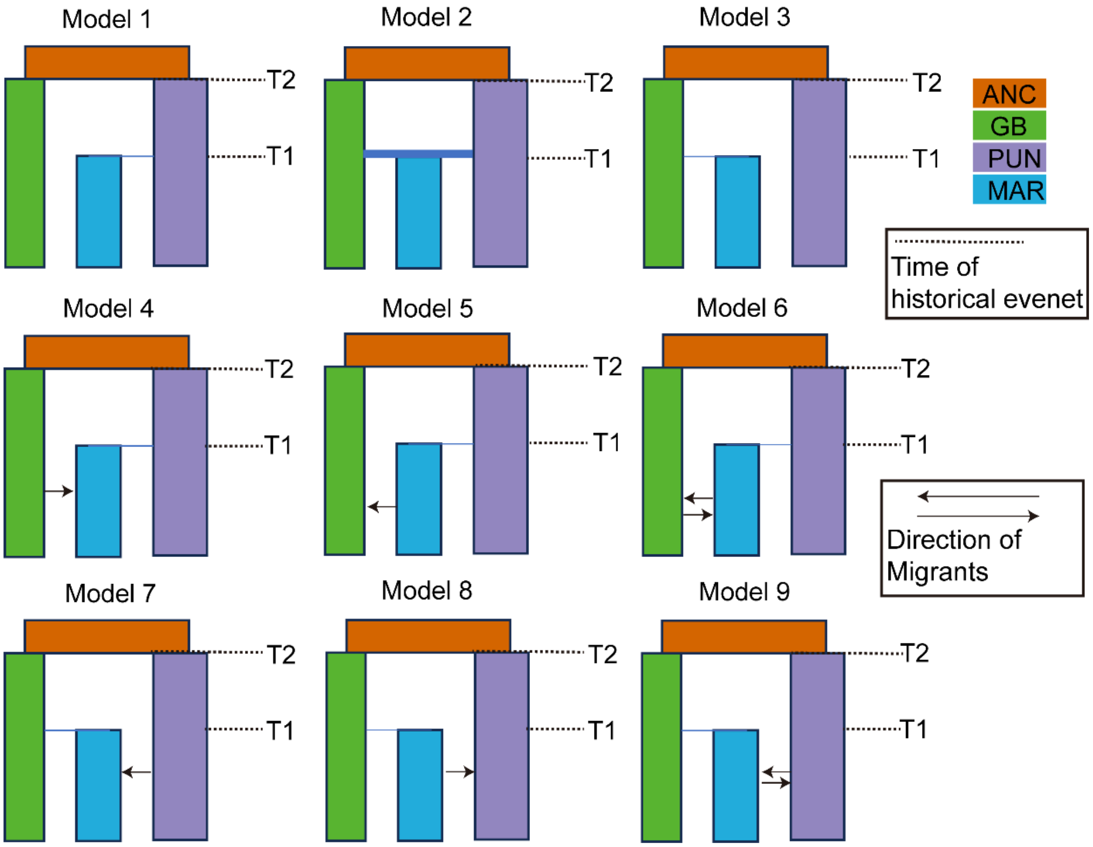

Fig. S19. Cartoons illustrate nine demographic scenarios for population divergence and the gene flow among PUN, GB and MAR groups.

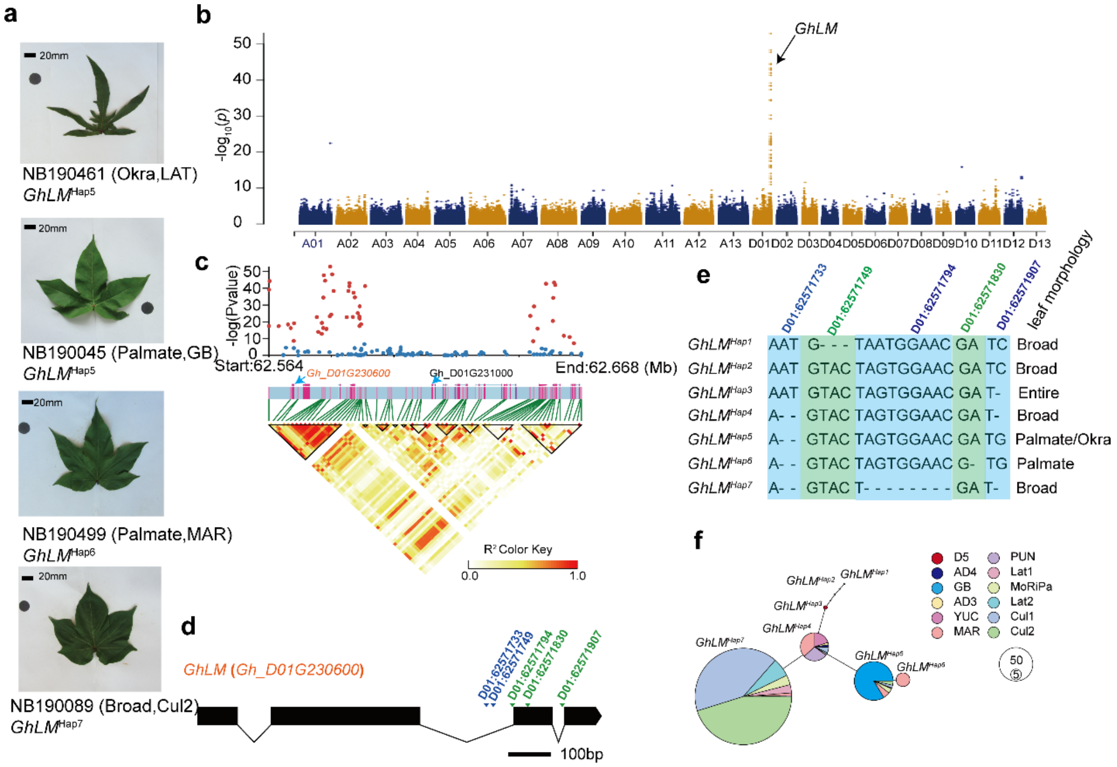

Fig. S20. GWAS for leaf morphology and identification of the candidate gene *GhLM*. a, Phenotypic features of leaf morphology in the following accessions. **b,** Manhattan showed the leaf morphology based on GWAS. **c,** Local Manhattan plot (top), gene distribution (middle), and local LD heatmap (bottom) around the peak associated with FM (leaf morphology). **d,** Gene structure and the variation site of *GhLM.* **e,** The major different haplotypes of *GhLM.* **f,** Haplotype network of seven haplotypes in 2,910 cotton accessions.

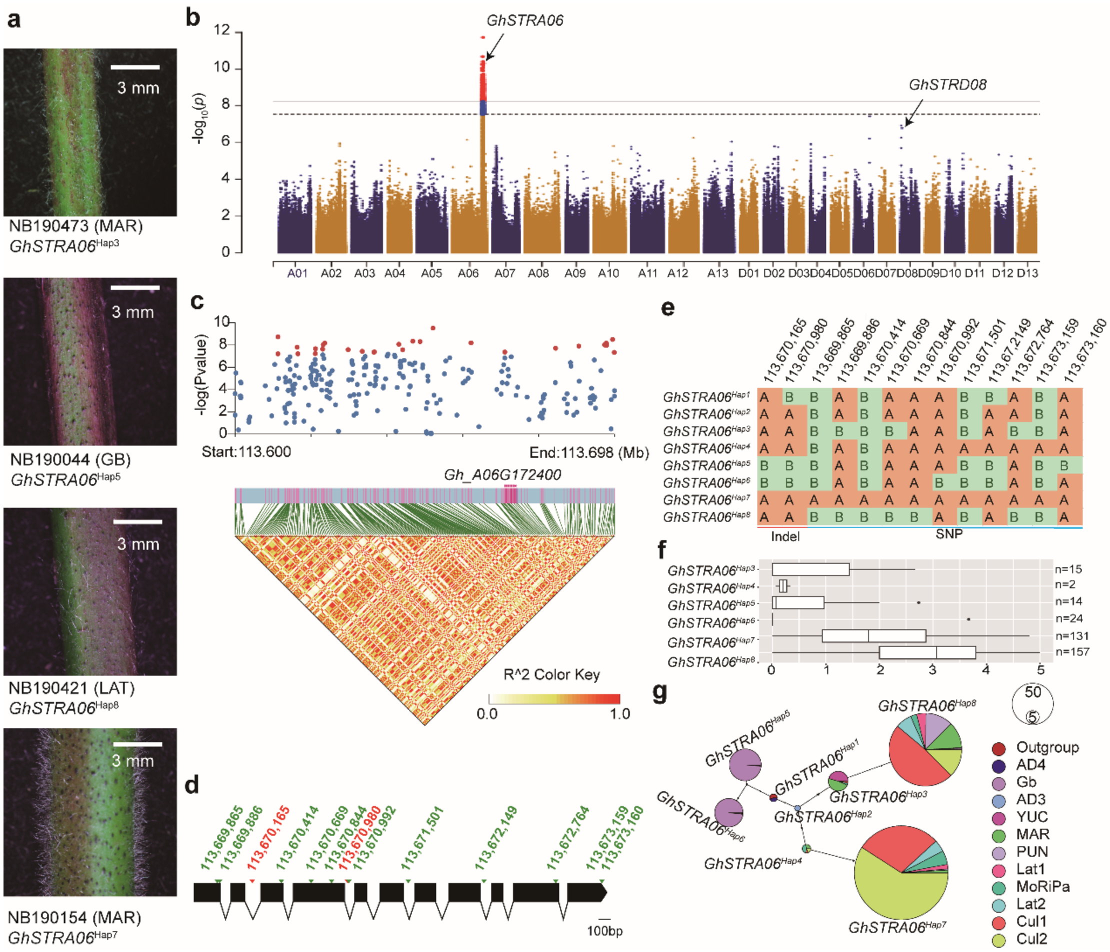

Fig. S21. GWAS for stem trichome and identification of the candidate gene *GhSTRA06*. a, Phenotypic features of stem trichome in the following accessions. **b**, Manhattan showed the stem trichome based on GWAS. **c,** Local Manhattan plot (top), gene distribution (middle), and local LD heatmap (bottom) around the peak associated with STR (stem trichome). **d,** Gene structure and the variation site of *GhSTRA06.* **e,** The major different haplotypes of *GhSTRA06.* **f,** The distribution of stem trichome traits for 5 haplotypes in *G. hirsutum*. **g,** Haplotype network of eight haplotypes in 2,910 cotton accessions.

References

1. Q. H. Zhu *et al.*, Integrated mapping and characterization of the gene underlying the okra leaf trait in L. *Journal of Experimental Botany* **67**, 763-774 (2016).
2. L. J. Chang *et al.*, Insights into Interspecific Hybridization Events in Allotetraploid Cotton Formation from Characterization of a Gene-Regulating Leaf Shape. *Genetics* **204**, 799-+ (2016).
3. D. Bates, M. Mächler, B. M. Bolker, S. C. Walker, Fitting Linear Mixed-Effects Models Using lme4. *Journal of Statistical Software* **67**, 1-48 (2015).
4. A. H. Paterson, C. L. Brubaker, J. F. Wendel, A rapid method for extraction of cotton (Gossypium spp.) genomic DNA suitable for RFLP or PCR analysis. *Plant Molecular Biology Reporter* **11**, 122-127 (1993).
5. D. Yuan *et al.*, Parallel and Intertwining Threads of Domestication in Allopolyploid Cotton. *Adv Sci* **8**, 2003634 (2021).
6. S. He *et al.*, The genomic basis of geographic differentiation and fiber improvement in cultivated cotton. *Nat Genet* 10.1038/s41588-021-00844-9 (2021).
7. Z. Yang *et al.*, Extensive intraspecific gene order and gene structural variations in upland cotton cultivars. *Nat Commun* **10**, 2989 (2019).
8. H. Li, Aligning sequence reads, clone sequences and assembly contigs with BWA-MEM. *ArXiv* **1303** (2013).
9. P. Danecek *et al.*, Twelve years of SAMtools and BCFtools. *Gigascience* **10** (2021).
10. A. Tarasov, A. J. Vilella, E. Cuppen, I. J. Nijman, P. Prins, Sambamba: fast processing of NGS alignment formats. *Bioinformatics* **31**, 2032-2034 (2015).
11. A. McKenna *et al.*, The Genome Analysis Toolkit: a MapReduce framework for analyzing next-generation DNA sequencing data. *Genome Res* **20**, 1297-1303 (2010).
12. S. Purcell *et al.*, PLINK: a tool set for whole-genome association and population-based linkage analyses. *Am J Hum Genet* **81**, 559-575 (2007).
13. P. Cingolani *et al.*, A program for annotating and predicting the effects of single nucleotide polymorphisms, SnpEff: SNPs in the genome of Drosophila melanogaster strain w1118; iso-2; iso-3. *Fly (Austin)* **6**, 80-92 (2012).
14. H. M. Kang *et al.*, Variance component model to account for sample structure in genome-wide association studies. *Nature Genetics* **42**, 348-U110 (2010).
15. S. S. Dong *et al.*, LDBlockShow: a fast and convenient tool for visualizing linkage disequilibrium and haplotype blocks based on variant call format files. *Briefings in Bioinformatics* **22** (2021).
16. S. McGinnis, T. L. Madden, BLAST: at the core of a powerful and diverse set of sequence analysis tools. *Nucleic acids research* **32**, W20-25 (2004).
17. W. Shen, S. Le, Y. Li, F. Hu, SeqKit: A Cross-Platform and Ultrafast Toolkit for FASTA/Q File Manipulation. *PLoS One* **11**, e0163962 (2016).
18. M. Lescot *et al.*, PlantCARE, a database of plant cis-acting regulatory elements and a portal to tools for in silico analysis of promoter sequences. *Nucleic acids research* **30**, 325-327 (2002).
19. L. T. Nguyen, H. A. Schmidt, A. von Haeseler, B. Q. Minh, IQ-TREE: a fast and effective stochastic algorithm for estimating maximum-likelihood phylogenies. *Mol Biol Evol* **32**, 268-274 (2015).
20. P. Danecek *et al.*, The variant call format and VCFtools. *Bioinformatics* **27**, 2156-2158 (2011).
21. D. H. Alexander, J. Novembre, K. Lange, Fast model-based estimation of ancestry in unrelated individuals. *Genome Res* **19**, 1655-1664 (2009).
22. C. Zhang, S.-S. Dong, J.-Y. Xu, W.-M. He, T.-L. Yang, PopLDdecay: a fast and effective tool for linkage disequilibrium decay analysis based on variant call format files. *Bioinformatics* **35**,

1786-1788 (2018).
23. J. Terhorst, J. A. Kamm, Y. S. Song, Robust and scalable inference of population history from hundreds of unphased whole genomes. *Nat Genet* **49**, 303-309 (2017).
24. L. Excofffier *et al.*, fastsimcoal2: demographic inference under complex evolutionary scenarios. *Bioinformatics* **37**, 4882-4885 (2021).
25. Y. Sun *et al.*, Population genomic analysis reveals domestication of cultivated rye from weedy rye. *Mol Plant* **15**, 552-561 (2022).
26. H. Chen, N. Patterson, D. Reich, Population differentiation as a test for selective sweeps. *Genome Res* **20**, 393-402 (2010).
27. A. R. Quinlan, BEDTools: The Swiss-Army Tool for Genome Feature Analysis. *Curr Protoc Bioinformatics* **47**, 11.12.11-11.12.34 (2014).
28. R. Verity *et al.*, minotaur: A platform for the analysis and visualization of multivariate results from genome scans with R Shiny. *Molecular Ecology Resources* **17**, 33-43 (2017).
29. J. D. Storey, R. Tibshirani, Statistical significance for genomewide studies. *Proc Natl Acad Sci U S A* **100**, 9440-9445 (2003).
30. S. Boitard, M. Boussaha, A. Capitan, D. Rocha, B. Servin, Uncovering Adaptation from Sequence Data: Lessons from Genome Resequencing of Four Cattle Breeds. *Genetics* **203**, 433-450 (2016).
31. S. H. Martin, J. W. Davey, C. D. Jiggins, Evaluating the Use of ABBA-BABA Statistics to Locate Introgressed Loci. *Molecular Biology and Evolution* **32**, 244-257 (2015).
32. D. Kim, B. Landmead, S. L. Salzberg, HISAT: a fast spliced aligner with low memory requirements. *Nat Methods* **12**, 357-U121 (2015).
33. Y. Liao, G. K. Smyth, W. Shi, featureCounts: an efficient general purpose program for assigning sequence reads to genomic features. *Bioinformatics* **30**, 923-930 (2014).
34. G. C. Yu, L. G. Wang, Y. Y. Han, Q. Y. He, ClusterProfiler: an R Package for comparing biological themes among gene clusters. *Omics* **16**, 284-287 (2012).
35. Y. F. Cai *et al.*, Genome sequencing of the Australian wild diploid species *Gossypium australe* highlights disease resistance and delayed gland morphogenesis. *Plant Biotechnology Journal* **18**, 814-828 (2020).
